# Investigation of Water Interaction with Polymer Matrices by Near-Infrared (NIR) Spectroscopy

**DOI:** 10.3390/molecules27185882

**Published:** 2022-09-10

**Authors:** Vanessa Moll, Krzysztof B. Beć, Justyna Grabska, Christian W. Huck

**Affiliations:** Institute of Analytical Chemistry and Radiochemistry, University of Innsbruck, 6020 Innsbruck, Austria

**Keywords:** near-infrared spectroscopy, NIR, polymer, water, polymer-water interaction, hydrophilic, hydrophobic, chemometrics, data analysis

## Abstract

The interaction of water with polymers is an intensively studied topic. Vibrational spectroscopy techniques, mid-infrared (MIR) and Raman, were often used to investigate the properties of water–polymer systems. On the other hand, relatively little attention has been given to the potential of using near-infrared (NIR) spectroscopy (12,500–4000 cm^−1^; 800–2500 nm) for exploring this problem. NIR spectroscopy delivers exclusive opportunities for the investigation of molecular structure and interactions. This technique derives information from overtones and combination bands, which provide unique insights into molecular interactions. It is also very well suited for the investigation of aqueous systems, as both the bands of water and the polymer can be reliably acquired in a range of concentrations in a more straightforward manner than it is possible with MIR spectroscopy. In this study, we applied NIR spectroscopy to investigate interactions of water with polymers of varying hydrophobicity: polytetrafluoroethylene (PTFE), polypropylene (PP), polystyrene (PS), polyvinylchloride (PVC), polyoxymethylene (POM), polyamide 6 (PA), lignin (Lig), chitin (Chi) and cellulose (Cell). Polymer–water mixtures in the concentration range of water between 1–10%(*w*/*w*) were investigated. Spectra analysis and interpretation were performed with the use of difference spectroscopy, Principal Component Analysis (PCA), Median Linkage Clustering (MLC), Partial Least Squares Regression (PLSR), Multivariate Curve Resolution Alternating Least Squares (MCR-ALS) and Two-Dimensional Correlation Spectroscopy (2D-COS). Additionally, from the obtained data, aquagrams were constructed and interpreted with aid of the conclusions drawn from the conventional approaches. We deepened insights into the problem of water bands obscuring compound-specific signals in the NIR spectrum, which is often a limiting factor in analytical applications. The study unveiled clearly visible trends in NIR spectra associated with the chemical nature of the polymer and its increasing hydrophilicity. We demonstrated that changes in the NIR spectrum of water are manifested even in the case of interaction with highly hydrophobic polymers (e.g., PTFE). Furthermore, the unveiled spectral patterns of water in the presence of different polymers were found to be dissimilar between the two major water bands in NIR spectrum (*ν*_s_ + *ν*_as_ and *ν*_as_ + *δ*).

## 1. Introduction

The interaction of water with different polymers has been an intensively studied research field [1,2,3], especially in recent years, with biocompatible polymers being one of the main focuses [4,5]. It has been demonstrated that the biocompatibility of a polymer is affected by its interaction with water [4]; furthermore, water–polymer interactions play a key role in biological processes [1,6]. The effect of moisture on commercially used polymers is also of high interest in material science and industrial applications. For example, an excess of water may cause swelling and, subsequently, changes of mechanical and chemical properties of polymers [1]. For these reasons, considerable attention has been diverted into investigations of the interaction of polymers with water, with a focus both on its phenomenological manifestations in various conditions as well as on its physicochemical background. With respect to the former, one of the promising concepts proposes to distinguish different species of water molecules in terms of their interaction strength with a polymer into strongly-bound, loosely-bound and free water species [1,4,6,7]. On the other hand, the so called “hydrophobic interactions” are often considered to be an important property of a material, appearing due to the interactions between water molecules being stronger than between water and the molecules of the hydrophobic material [8]. Hydrophobic interactions are highly dependent on various factors, e.g., temperature, size and shape of the interacting particles [8,9], among others. Insights into the underlying physicochemical properties of the interactions occurring between a polymer and water, including molecular structure effects, have been examined using various approaches. In these studies, diverse spectroscopic (e.g., vibrational, dielectric, nuclear magnetic resonance, etc. [7,10,11,12,13,14]) techniques, mass spectrometry [1], X-ray diffraction [15], differential scanning calorimetry [1,6,12] or gel-permeation chromatography [10] have been found to be helpful. Often, the experimental studies were combined with methods of computational chemistry to provide deepened physical insights [16,17].

Vibrational spectroscopic techniques, MIR and Raman, were often used to derive both phenomenological and molecular insights into the effects of the interactions between water and polymers [12,14,18,19,20]. In contrast, NIR spectroscopy has not yet attracted similar attention in the studies of this problem. This spectroscopic technique offers unique suitability for this purpose [21,22], as the intensity change in water absorption is known to mirror the change in the chemical environment of water molecules [11,23]. Spectral bands in NIR spectroscopy manifest unique sensitivity towards the chemical environment and hydrogen bonding [21,24,25]. The positions and intensities of NIR bands, primarily arising from combinations and overtones of C-H, O-H and N-H stretching vibrations, are intrinsically related to the properties of hydrogen bonding existing in the investigated system [12,13]. Because of the profound influence of specific interactions on mechanical and electrical anharmonicity of the partner molecules [26], NIR spectra provide information on the properties of hydrogen-bonded complexes that is unavailable in MIR or Raman spectra [27]. Consequently, NIR spectroscopy provides exclusive opportunities for the investigation of molecular structure and interactions [21,25]. These effects manifested in NIR spectra can be utilized to investigate the interaction of the hydrogen-bonding centers, present in the polymer, with water and provide insight into the interaction behavior of these species [11,21,25]. Therefore, NIR spectroscopy has been demonstrated to provide valuable information for the characterization of polymers and their composites [22,28].

Physical principles underlying NIR spectroscopy make it also very well suited for the analysis of aqueous systems in a practical sense. NIR bands of water feature relatively weaker intensities, in contrast to very strong bands of water in the MIR region [21,29]. This makes it much easier to examine both the bands of water and the polymer in the NIR spectra, particularly over a wider range of water concentrations in the sample [21]. Although less of a critical hindrance than it appears in MIR spectroscopy, the water bands in NIR spectra can still obscure (i.e., mask) the signal of other constituents present in the sample [21,23]. In certain applications this remains to be an unwanted effect, for which developing effective mitigation methods would be helpful. Even though the removal of water bands from vibrational spectra has been studied for years, there is still very little knowledge of universal reach gathered in this area. This specific problem was almost exclusively investigated using the MIR technique [29,30]. A considerable focus has been directed at the suppression of the ro-vibrational structure of water vapor, as atmospheric water is the source of a common interference in MIR spectroscopy. The need for effective removal of water bands was identified relatively early in the field of the applications of NIR spectroscopy, with most of the proposed approaches to alleviate this problem being chemometric methods [23,31,32] and wavenumber selection methods [33]. Some attempts were made by using the refinement [23,31] of the Orthogonal Signal Correction method [34]. For example, the Regional Orthogonal Signal Correction was one of the approaches proposed, in combination with Moving Window Partial Least Squares Regression, to remove interfering water signals from NIR spectra [23]. Other well-known spectral transformation techniques were also evaluated for this purpose. For the investigation of the phosphorus and nitrogen concentration in fresh leaves [32], a non-linear Least Squares Spectral Matching technique was introduced [35], where the spectrum of a fresh leave was approximated by a nonlinear combination of the leaf-water spectrum and a dry sample spectrum. Nevertheless, no practically applicable method of universal reach could be established, due to major limitations in the transferability to other data sets, accuracy, overfitting [32] and noteworthy complexity for the user, because individual calculations and sample-tailored solutions were necessary for each specific case. Owing to single, purpose-driven NIR spectroscopic studies of these effects, the knowledge gathered so far remains fragmentary; little attention has been given to systematic studies of series of compounds of relatively similar character but with gradually varying key properties affecting their interaction with water.

In this study, we investigated polymer–water interactions and the manifestation of this phenomenon in NIR spectra by applying a systematic approach and employing a synergistic set of methods and techniques. We attempted to provide a more universal reach and deeper insights into the problem of water bands obscuring the signal of the analyzed compound in NIR spectra. For this purpose, polymers of varying hydrophilicity were investigated by diffuse reflectance NIR measurements: polytetrafluoroethylene (PTFE), polypropylene (PP), polystyrene (PS), polyvinylchloride (PVC), polyoxymethylene (POM), polyamide 6 (PA), lignin (Lig), chitin (Chi) and cellulose (Cell). Pure polymers as well as polymer–water mixtures in the concentration range of 1–10% (*w*/*w*) of water were analyzed. Spectra analysis and interpretation were performed with the use of difference spectroscopy, Principal Component Analysis (PCA), Median Linkage Clustering (MLC), Partial Least Squares Regression (PLSR), Multivariate Curve Resolution Alternating Least Squares (MCR-ALS) and Two-Dimensional Correlation Spectroscopy (2D-COS). Additionally, from the obtained data, aquagrams were constructed and interpreted with aid of the conclusions drawn from the conventional approaches. By simultaneous use of synergistic tools, generalized trends in the spectral manifestation of the interaction of water with polymers, including the dependencies on chemical nature and hydrophobicity, were obtained. In addition to physicochemical insights, these conclusions provide better understanding of the effects of water–solid matrix interactions, which often play a meaningful role in various applications of NIR spectroscopy.

## 2. Materials and Methods

### 2.1. Samples and Data Aquisition

#### 2.1.1. Polymer Samples

The polymer samples were acquired as standards for synthetic, non-water-soluble, polymers from the suppliers present at the commercial market (Saudi Basic Industries Corporation SABIC, INOVYN, INEOS Styrolution, Euro OTC Pharmas GmbH, Sigma Aldrich). Cellulose (synthetic), lignin (kraft), chitin (from shrimp shells), PTFE and PVC were derived as practical grade powder, with an approximate particle size of 100 µm. PP, PS, PA and POM samples were acquired as pellets from different manufacturers. The polymer pellets were separately milled with the centrifugal mill ZM 200 (Retsch, Verder Scientific, Haan, Germany) while being cooled with liquid nitrogen to prevent temperature-induced changes. The centrifugal mill was equipped with a sieve with the pore size selected to obtain the particle diameter of approximately 250 µm. Deionized water was prepared by a Milli-Q^®^ Reference (Merck KGaA, Darmstadt, Germany), with a conductance of 18.2 MΩcm. To ensure reproducibility, the polymer powders were completely dried in the drying chamber, at 50 °C and with a pressure of 200 mbar. An hour before measuring, the polymers were equilibrated to room temperature and stored in a desiccator until measurement. 

#### 2.1.2. NIRFlex N-500 FT-NIR Spectrometer 

Measurements were performed with the NIRFlex N-500 FT-NIR spectrometer (BÜCHI Labortechnik AG, Flawil, Switzerland) with the attachment for solid sample measurements and a spinner add-on, which enables spatial averaging of the sample spot during the spectra measurement. The NIRFlex N-500 is equipped with a HeNe laser as a high-precision wavelength reference, a polarization interferometer with TeO_2_ wedges and a tungsten halogen lamp for sample irradiation. Measurements were performed in diffuse reflection mode; 64 scans were accumulated per single spectrum, with an optical resolution of 8 cm^−1^, in the wavenumber region of 10,000–4000 cm^−1^. Cylindrical cuvettes for reflection measurements of solid samples, made of optical glass, with a volume of approximately 12 mL, were purchased from Hellma (Müllheim, Germany).

#### 2.1.3. Data Acquisition 

All polymers were directly weighted and prepared in the measuring cells. The amount of each individual polymer was constant throughout all measurements. Respectively, 1%, 3%, 5%, 7% or 10% deionized water (*w*/*w*) was added. Afterwards, the polymer–water mixtures were stirred for approximately 165 s with disposal spatulas, to ensure homogenous distribution of water in the polymer matrices. A metal stamp with a Teflon-foil ring was used to seal the measuring cells, to prevent water evaporation and ensure constant measurement conditions, by pressing the polymer–water mixtures to the ground of the cuvettes. The preparation of the samples and their placement in the measurement cell was repeated six times for each polymer–water mixture and each concentration level, in order to monitor the reproducibility of the procedure; the spectra measurements were done in triplicate. This procedure was performed for all polymers, with the exception of PTFE. Since PTFE is highly hydrophobic, it repels water completely and is not mixable with water at all. Therefore, we measured nine spectra of PTFE, with approximately 10% of water (water was the bottom layer). These spectra were then averaged, in order to overcome the variances in spectral intensity due to variation of the thickness of the water layer. At this stage, PLSR analysis was used to identify outliers in the measured spectral dataset; for the identified sample outliers, the measurements were repeated.

### 2.2. Chemometric Methods–Spectra Processing and Analysis

The collected raw spectra were transferred into the Unscrambler^®^ X Version 10.5 (CAMO Software, Oslo, Norway). Before spectral analysis, firstly the spectra were recalculated from reflectance R into absorbance A, by applying a negative common logarithm (log 1/R). A linear offset correction was then used as a pretreatment method; it enables direct comparison of all measurements and polymers. For most of the analysis methods, the spectral dataset was averaged to one spectrum per concentration, except for PCA and PLSR analysis, where no sample averaging was used. All plots were generated with OriginPro^®^ 2020. Noteworthily, the spectra below 4500 cm^−1^ should be considered less reliable, as the complete absorption phenomenon occurred for several samples. However, this region was not used for the purpose of this study, nor are any discussions in this work based on this fragment of spectra. Nonetheless, throughout this manuscript, full spectral data are presented (i.e., in the region of 10,000–4000 cm^−1^), as they may be found useful by the readers for qualitative (i.e., rough) assessment.

#### 2.2.1. Principal Component Analysis (PCA) and Median Linkage Clustering (MLC)

PCA and MLC were performed with the Unscrambler^®^ X Version 10.5. The polymer–water mixture spectra, pretreated by linear offset correction, were used for this purpose. Full-cross-validation by means of the leave-one-out (LOO) approach was performed, and for determining the latent variables in the PCA approach, a nonlinear iterative partial least squares algorithm (NIPALS) was utilized. As the MLC method, a hierarchical clustering with a squared Euclidean distance measurement and the number of eight clusters (corresponding to the eight polymers used in this study), was used.

#### 2.2.2. Partial Least Squares Regression (PLSR)

PLSR was carried out with the Unscrambler^®^ X Version 10.5. The linear offset was applied to correct and normalize the spectra prior the generation of the PLSR models. A full-cross-validation by means of the LOO approach was conducted, and an NIPALS algorithm was used for determining the latent variables in the PLSR procedure.

#### 2.2.3. Difference Spectroscopy

Difference spectroscopy was conducted manually; all calculations were carried out with Microsoft^®^ 365 Excel^®^. For this purpose, the averaged, linear offset corrected spectra were used. The polymer difference spectra were generated by firstly scaling the water spectrum individually for each polymer–water mixture spectrum. The peak maximum of the combination water band, located at 5180 cm^−1^, was utilized as the scaling reference point. At 5180 cm^−1^, the intensity of the water band was scaled to the intensity of the water peak in each polymer–water mixture spectra, by dividing the intensity of the sample spectra by the intensity of the pure water spectrum. The scaling factor generated this way was used to multiply the water spectrum at each wavelength, which subsequently was subtracted from the respective polymer–water mixture spectrum. The water difference spectra were generated in an analogous procedure, by subtraction of the pure polymer spectrum from the mixture spectra, with individual scaling wavelengths for each polymer. The following reference points in the spectra of polymers were selected for this purpose: PTFE at 5944 cm^−1^ PP at 5796 cm^−1^, PS at 5952 cm^−1^, PVC at 5828 cm^−1^, POM at 5968 cm^−1^, PA at 5828 cm^−1^, lignin at 5964 cm^−1^, chitin at 5800 cm^−1^ and cellulose at 5604 cm^−1^. 

#### 2.2.4. Multivariate Curve Resolution Alternating Least Squares (MCR-ALS)

A multivariate curve resolution (MCR) analysis was performed with the Unscrambler^®^ X Version 10.5. The polymer–water spectra pretreated by linear offset correction were used, and the averaged pure water and polymer spectra were provided as a Y-reference. Two components were selected in this procedure to match the chemical rank of binary mixtures. Constraints were set to non-negativity for concentrations and spectra. The MCR procedure was performed using an alternating least squares algorithm (i.e., MCR-ALS). In order to compare the resulting spectra with the experimental gathered spectra, a SNV transformation had to be performed on both spectra sets. 

#### 2.2.5. Two-Dimensional Correlation Spectroscopy (2D-COS)

A 2D-COS analysis was accomplished using the extension 2D Correlation Spectroscopy Analysis, available in OriginPro^®^ 2020. This software enables calculation of synchronous and asynchronous 2D-COS spectra. The averaged spectra of the pure polymer and the polymer–water mixture spectra were selected as dynamic spectra, with the concentrations as perturbations. The average dynamic spectrum was used as the reference. Subsequently, the synchronous and asynchronous 2D-COS plots were calculated for all polymers. Note, for better comparison, in this work, the synchronous plots are presented in an identical scale of intensity (*z*-axis) for all polymers. The intensity axis was chosen in a way so that all relevant information is easily accessible and the correlation strength is directly comparable. The intensities of the asynchronous plots on the other hand are scaled individually, because the intensity ranges are much less comparable between different systems, and uniform scaling would compromise the accessibility to the individual information on each sample.

#### 2.2.6. Aquagrams

Aquagrams generally display water patterns exclusively for the overtone water band, which is not comprehensive enough to describe the dissimilarities of the investigated polymer–water systems. Therefore, aquagrams in this study were expanded to include also the second major water band, the combination band. Wavelengths of interest were selected by a comparison of all normalized polymer spectra. For the normalization, the averaged polymer–water mixture spectra were used. Firstly, an SNV transformation was performed in the Unscrambler^®^ X Version 10.5 as pretreatment, and afterwards, the normalized absorbance $A^{n}{}_{\lambda }$ for each spectrum and, respectively, each polymer was calculated regarding Equation (1) in Microsoft^®^ 365 Excel^®^. Where $A_{\lambda }$ is the SNV transformed absorption spectrum, ${µ}_{\lambda }$ is the mean spectrum of the regarding polymer and $\sigma_{\lambda }$ is the standard deviation for the regarding polymer spectra after SNV transformation [36]. The selected wavenumbers were consequently plotted in an extended aquagram, representing both water bands.

(1) $$A^{n}{}_{\lambda }=\frac{A_{\lambda }-{µ}_{\lambda }}{\sigma_{\lambda }}$$

## 3. Results

### 3.1. General Features of the NIR Spectra of Polymer–Water Systems

**Figure 1:**
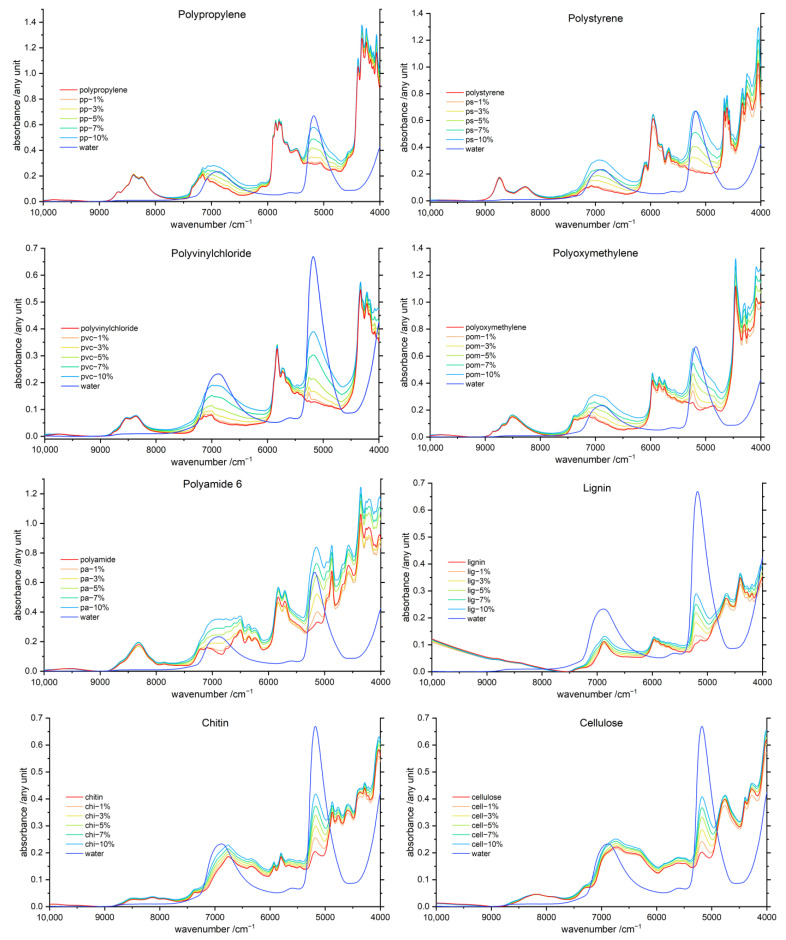
Averaged NIR absorbance spectra of the polymer–water mixtures after linear offset correction, in the range of 1–10% (*w*/*w*) water and the pure water spectra (dark blue) for comparison. The polymers are ordered according to increasing hydrophilicity, with the least hydrophilic polymer, polypropylene (**upper left** corner), to the most hydrophilic polymer, cellulose (**lower right** corner).

The averaged NIR spectra of the polymer–water mixtures, additionally corrected by applying a linear offset of the baseline, are displayed in Figure 1. Note, the two major NIR bands of water have a complex internal structure, resulting from overlapping contributions from different species, and their exact nature is a matter of intensive and long-lasting discussions [37,38]. These bands originate primarily from combination vibrations, respectively, *ν*_s_ + *ν*_as_ in the case of the peak observed at ca. 6900 cm^−1^ and *ν*_as_ + *δ* for the band at ca. 5200 cm^−1^. However, in the case of the former one, a meaningful component of the OH stretching overtone (2*ν*_OH_) is present as well. Despite that contribution to the intensity being lesser, it is commonly accepted in literature to refer to the ca. 6900 cm^−1^ band of water as the “overtone band”. The band observed at ca. 5200 cm^−1^ is described as the “combination band”, which precisely reflects its nature. For clarity, that commonly accepted, albeit not entirely precise, naming convention for those spectral features will be adopted in this work. 

In Figure 1, spectra of the samples containing a varying water content are clearly differentiable for all polymers, with some variances manifested in the spectra of dissimilar polymers, which can be easily noticed. Interestingly, it can be noted that the variation in water content also influences the intensity of the polymer bands. This effect tends to grow with rising hydrophilicity for all investigated materials. Furthermore, it is not suppressed upon performing a linear offset correction or Standard Normal Variate (SNV) treatments, indicating that polymer–water interactions may be responsible for these intensity variations. In general, the biopolymers notably differ from the synthetic polymers, as they show more constant changes in the NIR spectra of the samples with varying water content. Moreover, clear red- and blueshifts of the water bands for different polymers and water concentrations are observed. In the case of hydrophobic polymers, the appearance of the spectra is distinctly influenced by the amount of added water. One the one hand, a low water content in the polymer matrix leads to strongly shifted and deformed water bands. On the other hand, when more water is added to the sample, the appearance of the water bands gets less deviated from that of bulk water. For example, PP shows a pronounced shift of the combination band of water for the sample spectrum containing 1% of water. On the contrary, the spectrum of the sample containing 10% water reveals a water band at the position very similar to that of bulk water. 

However, hydrophobic polymers are anticipated to only weakly interact with water. Indicating, that only a small amount of water interacts with the polymer, and the addition of more water results in the presence of free bulk water. This is also supported by the finding that water band shifts get more uniform with increasing hydrophilicity of the samples. The water bands in the presence of biopolymers show almost completely constant shifts through all concentration levels. Interestingly, both major water bands show a dissimilar behavior in the presence of different polymers. For instance, blue- and redshifts for the same sample are manifested in the NIR spectra, e.g., for PA, a blueshift is observable for the overtone band of water, whereas a slight redshift is present for the combination band. Moreover, wavenumber shifts are much more pronounced for the combination band than for the overtone water band.

### 3.2. Band Assignment

As presented in Figure 1, most of the polymers show strong and specific polymer bands in the wavenumber region of 9000–8000 cm^−1^, between 7500–7000 cm^−1^, in the region of 6500–5500 cm^−1^ and near 4500 cm^−1^. Additionally, more hydrophilic polymers show peaks in the vicinity of both major water bands. Especially for the combination band of water, the polymer spectra reveal a signal growing in intensity with rising hydrophilicity, which most likely indicates the presence of trace water bound to hydrophilic polymers, even for dried samples. Biopolymers are highly hydrophilic and therefore always contain bound water [11]. The overtone band of water displays a peak maximum at approximately 6900 cm^−1^ in this case, even though both water bands arise due to combinations of vibrational modes. In Table 1, we provided the assignments for the major polymer and water vibrations.

### 3.3. Principal Component Analysis (PCA) and Median Linkage Clustering (MLC)

**Figure 2:**
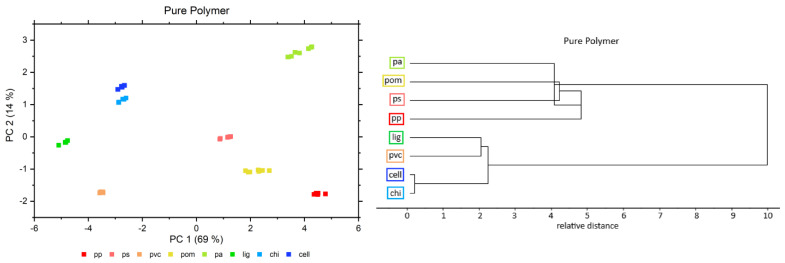
PCA scores (**left**) and MLC dendrogram (**right**) for the pure polymers after linear offset correction. The PCA scores and MLC dendrograms for the entire concentration range of water (1–10%) added to the polymer (*w*/*w*) are displayed in the Supplementary Materials (Figures S1 and S2, respectively).

A PCA and a hierarchical MLC method were utilized for a general inspection of the spectral set and analysis of the distribution of the samples to verify the consistency of the experimental conditions. Furthermore, these methods also enabled us to gain an overview of the trend related to the polymer hydrophilicity in the samples containing different concentrations of water. For this purpose, a PCA and a hierarchical MLC were respectively performed for the pure polymers, as well as for each individual concentration level of water in polymer –water samples. Exemplary PCA scores and an MLC dendrogram for the pure polymers are illustrated in Figure 2. The figures presenting the PCA score plots and MLC dendrograms for the entire concentration range of water (1–10%) added to the polymer (*w*/*w*) are displayed in the Supplementary Material (Figures S1 and S2, respectively). 

The PCA scores in Figure 2 reveal perfectly separated groups for each individual polymer, without greater variance in between the repetition measurements of a single polymer. Cellulose and chitin are aligned relatively close to each other, but still, both polymers are easily differentiable. This reflects the high similarity of cellulose and chitin, which only differ in one functional group. Additionally, lignin is located near to chitin and cellulose, which may be interpreted as the relatively greater similarity of the biopolymers in comparison to all other polymers. The comparison of the PCA scores for the pure polymers and the water–polymer systems with 1–10% water (*w*/*w*) revealed no significant changes in the distribution of the samples, as presented in Figure S1. 

The MLC analysis revealed the presence of two major groups in between the investigated polymers (Figure 2 and Figure S2). Interestingly, the first major cluster consisted of three biopolymers and PVC, with chitin and cellulose forming a subcluster and lignin and PVC another subcluster. The second major cluster includes the remaining synthetic polymers. This grouping corresponds well to the PCA scores plotted in Figure 2. 

### 3.4. Partial Least Squares Regression (PLSR)

**Figure 3:**
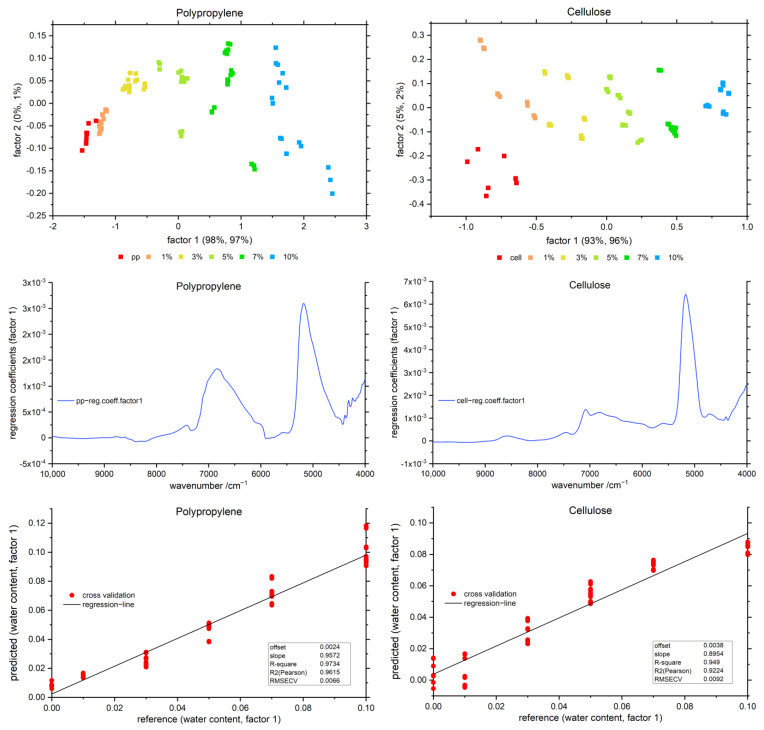
PLSR scores (**top**), regression coefficients (**middle**) and predicted vs. reference (**bottom**) for polypropylene (**left**) and cellulose (**right**) polymer–water mixtures, after linear offset correction. The scores, regression coefficients and predicted vs. reference of all polymers in comparison are displayed in the Supplementary Materials (Figures S3–S5).

A PLSR was performed for all samples in order to validate that the observed spectral variations were indeed well-correlated with the concentration of water in the sample. On the example of PP and cellulose, the resulting scores, regression coefficients for factor 1 and prediction performances of the cross-validation are displayed in Figure 3. The PLSR metrics obtained for all polymers investigated in this study and the regression coefficients for factor 1–3 are provided in the Supplementary Material (Figures S3–S5). 

A clear separation of the different water contents and the pure polymers can be observed in the scores plots in Figure 3. Minor tendencies for sample clustering are apparent and should be accounted to the variations in the sample preparation process or unavoidable external conditions, e.g., the temperature and humidity. However, these effects are nearly negligible and not expected to interfere with the main investigation of this study. For all polymers, a high quality of the model fit was obtained in the PLSR procedure; an R^2^ of at least 0.93 or higher was obtained in each case. This clearly indicates that the water concentration levels manifested in the NIR spectra were indeed near the nominal values intended for the prepared sample. No other effects of random or polymer-specific character, resulting, for example, from a potential vaporization or different distribution of liquid water in granulated polymer matrix, occurred in the sample set that could introduce spectral changes other than those directly correlated with water concentration. For all polymers within the first two factors, at least 98% of the variation in the NIR spectra was explained by 98% of the variation in the water concentration. Interestingly, the regression coefficients also showed resemblance to the water spectrum itself, conforming that water is the main inductor for changes in the spectra and for grouping of the samples in the scores plot (Figure 3). 

### 3.5. Difference Spectroscopy

#### 3.5.1. Water difference Spectra

**Figure 4:**
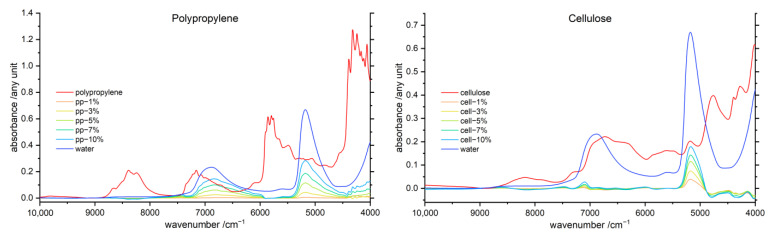
Water difference spectra of the polymer–water mixtures after linear offset correction and subtraction of the polymer spectra, in the range of 1–10% (*w*/*w*), with, respectively, the pure water (dark blue) and polymer (red) spectra for comparison, of PP (**left**) and cellulose (**right**). The water difference spectra of all investigated polymers are displayed in the Supplementary Materials (Figure S6).

A difference spectroscopy approach was applied to elucidate the NIR line shape of the water component present in the samples. In the procedure, the spectra of the pure polymers were subtracted from the spectra of polymer–water mixtures after the treatments to normalize spectral sets were applied as described in Section 2.2.3. In Figure 4, the line shapes resolved for the water component in the presence of PP and cellulose are displayed, while the results of this procedure for the remaining six polymer–water systems are provided in the Supplementary Materials (Figure S6).

The water difference spectra revealed significantly different shapes of the water bands for each individual polymer. However, the extent of band change neither followed hydrophilicity, nor was it related to the chemical nature of the polymer, indicating that another effect was in play causing the observed specificity. Interestingly, the water spectrum of the 1% PP–water mixture in Figure 4 is nearly featureless, with only a very shallow and broad peak at the combination water band; this is also noticeable in the raw spectrum in Figure 1. Furthermore, Figure S6 reveals that the water bands arose only with rising hydrophilicity of the polymer matrix. This effect was clearly present in the spectra and related to the polymer hydrophilicity. Interestingly, these spectra evidence the presence of strongly bonded water molecules observed in hydrophobic matrices such as PP (specifically, steadily increasing intensity and broadened shape of both water bands). However, the spectra of the systems involving hydrophilic polymers such as cellulose reveal that, rather, weakly interacting water species are present in such matrices at low concentrations (specifically, narrow, blue-shifted overtone band of water at ca. 7100 cm^−1^). This observation suggests that the formation of strongly-interacting bulk-like water domains is promoted in hydrophobic matrices such as PP. At the same time, in the cellulose matrix, apparently the formation of bulk-like water is not promoted at low concentrations. This might occur because the hydrophilic matrix attracts more water molecules than hydrophobic surfaces of polymers such as PP. Consequently, a hydrophilic matrix creates a more competitive environment for binding water molecules, and bulk-like water domains are less easily formed at very low concentration of water in the matrix.

A separate note should be made about the inconsistency of the intensity change observed between the water overtone and combination band in the cellulose matrix being not uniform. The intensity of the overtone band with water concentration increases less rapidly than it is observed for the combination band. This seems to be plausible, as the electrical anharmonicity of hydrogen-bonded species has a profound effect in the intensities of overtone bands [26,27].

Moreover, PP, PS, PVC and POM reveal a highly specific behavior, with the water bands being profoundly asymmetric. For these systems, the presence of differently interacting water species is manifested in the NIR spectra. On the one hand, the water molecules weakly interacting with moderately hydrophilic polymer can be identified by the appearance of a water band for the combination band and overtone band. Furthermore, the existence of a broadened absorption feature extending towards lower wavenumbers (i.e., a broad band shoulder) reveals the presence of self-interacting water, i.e., bulk-like water domains. In the case of PP and PS, the revealed water bands are significantly widened, together with the additional extension towards lower wavenumbers; this indicates the presence of two different bulk-like water domains. This suggests that these polymer matrices effectively create two different chemical environments for water molecules. It is also possible that physical properties and morphology of the particles of these polymers are in play here; for instance, the less-developed areas of the hydrophobic surface of these polymers might lead to a faster evaporation of water from the polymer surface. Surprisingly, the lignin–water system also reveals a pronounced water band component observed at the low wavenumber shoulder of the combination water band. This suggests that lignin only weakly interacts with water, which promotes the organization of self-associated domains of water resembling bulk water. Noteworthily, the PA–water system interrupts this trend, which might be stemming from the chemical nature of this polymer. On the other hand, the water spectra for chitin and cellulose are relatively uniform. These effects can be observed for both water bands, and therefore, polymer–water interactions are strongly manifested in the NIR spectra. Note, in the case of the lignin–water system, the overtone water band is distorted by the subtraction procedure, and therefore, the water component of this sample should be considered less reliable.

Furthermore, distinct wavenumber shifts of both water bands occur in the presence of different polymers. The shift is especially noticeable for the overtone water band; the respective band shifts for each polymer are listed in Table 2. The biopolymers show, in this wavenumber region, profoundly broadened and strongly shifted water bands. 

#### 3.5.2. Polymer Difference Spectra

**Figure 5:**
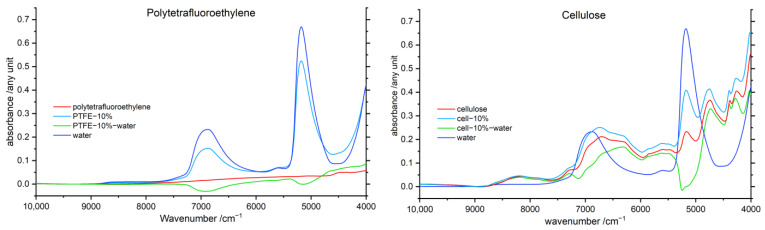
Polymer difference spectra of the 10% polymer–water mixtures after linear offset correction and subtraction of the water spectrum, with, respectively, the pure water (dark blue) and polymer (red) spectra for comparison of PTFE (**left**) and cellulose (**right**).

With the aim to elucidate the variations in NIR spectra of the polymers, which can potentially occur as the effect of the interaction with water, the difference spectroscopy approach was applied as well to resolve the line shape associated with the polymer component. In this case, the spectrum of pure water was subtracted from the spectra of the water–polymer samples in the subtraction procedure. PTFE was the most hydrophobic polymer included in our study; the interaction between water and PTFE should be distinctively low. Furthermore, it has no meaningful absorption in the NIR region. Therefore, PTFE offers favorable properties for the validation of the use of difference spectroscopy in this study (Figure 5). The figure additionally displays the difference spectra of the cellulose–water system, as it constitutes the most hydrophilic polymer examined in this study.

The resolved polymer component spectrum of the averaged 10% PTFE–water system in Figure 5 shows two broad negative features in the wavelength region of both water bands. A similar result was obtained for cellulose but with even more pronounced adverse features. The most probable reason of this is the presence of several OH groups in cellulose and likely also the relatively higher content of strongly bound inherent water molecules persisting in dried cellulose. Therefore, cellulose is highly interacting with water molecules. In NIR spectra, hydrogen-bonded species feature lower band intensities [26,27]; consequently, the spectrum of water bound strongly to cellulose differs from that of bulk water. This effect in combination with the dissimilar behavior of both major water bands, described in Section 3.1, confines the applicability of the polymer difference spectroscopy notably. Because of these limitations, the MCR-ALS study (Section 3.6) was conducted to provide independent, and potentially less affected by imperfections of the method itself, insights into the components of the NIR spectra associated with each of the interacting species. On the other hand, the results of difference spectroscopy clearly evidence the manifestation of polymer–water interactions in the spectra, even for highly hydrophobic polymers, i.e., PTFE. Therefore, for effectively revealing NIR peaks of the polymer masked by water bands, the polymer–water interactions should be considered. Especially biopolymers or other plant materials strongly interact with water. These highly hydrophilic and potentially hygroscopic materials always contain water by nature.

### 3.6. Multivariate Curve Resolution Alternating Least Squares (MCR-ALS) 

**Figure 6:**
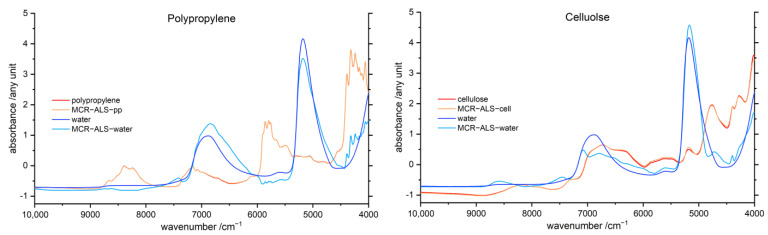
MCR-ALS polymer (orange) and water spectra (light blue) of polypropylene (**left**) and cellulose (**right**), additionally the NIR absorbance spectra of the pure polymer (red) and pure water (dark blue) are shown. The reference spectra as well as the resolved curves were normalized using an SNV transformation. The MCR-ALS polymer and water spectra of the remaining six polymers are displayed in the Supplementary Materials (Figure S7).

An MCR-ALS analysis provides decomposition of the polymer–water mixture spectra into the resolved spectral curves associated with each of the components, i.e., in this case, water and the polymer spectra. The resolved curves are presented in Figure 6 for PP and cellulose, while the results for all eight investigated polymers are provided in the Supplementary Materials (Figure S7).

In general, the MCR-ALS polymer spectra are very similar to the experimental spectra measured for the pure polymers, indicating physical representativeness of the resolved curves. Consequently, the resolved spectral curve of the water component accurately reflects the true absorption profile of water existing in polymer matrix. In the case of PP, the resolved polymer component is almost undistinguishable from the spectrum measured for the pure polymer. In the case of the remaining polymers, the MCR-ALS curves show some minor deviations, almost exclusively located in the wavenumber regions of both water bands. However, these deviations form a trend. Especially in the vicinity of the combination water band, the resolved curves reveal a water band growing in intensity with rising hydrophilicity of the polymer. Noteworthy, for highly hydrophilic chitin and cellulose, the MCR-ALS spectra are surprisingly similar to the experimental spectra of pure polymers. The highest changes are obtainable for the hydrophilic polymers POM, PA and lignin. In contrast, the resolved water component curves for both water bands highly diverge from the experimental NIR spectrum of bulk water. Moreover, significant changes in band-shape and additionally band shifts are observed. While the absolute band intensities of the resolved MCR-ALS line shapes are not representative because of SNV treatment, the analysis of the intensities of the two major water bands remains legitimate in relative sense. Interestingly, the resolved water bands indicate diminished intensity of the combination band and enhanced intensity of the overtone band of water in comparison with those of bulk water for synthetic polymers. However, for the biopolymer matrices, an opposite trend in relative intensities of water bands can be noticed. 

Furthermore, in the resolved water curves, the overtone band diverges (in shape and position of peak maximum) more substantially from the experimental water spectrum than it occurs for the combination band of water. In the case of lignin and cellulose, the resolved component spectra may be considered less reliable, because a splitting of the overtone MCR-ALS water band into two peaks was observed. Analogous to the water difference spectra discussed in Section 3.5.1, for PP, PS, PVC and POM, a low wavenumber shoulder of the water band component was revealed for the water overtone and combination band, indicating strong interactions between the water and polymer matrix. Therefore, the presence of both strongly and weakly interacting water can be evidenced from the MCR-ALS water curves.

### 3.7. Two-Dimensional Correlation Spectroscopy (2D-COS)

**Figure 7:**
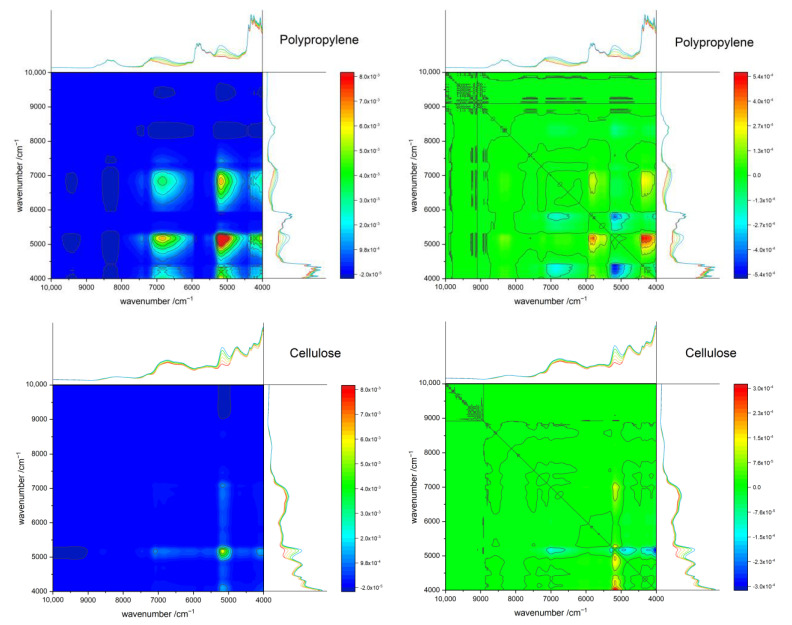
Synchronous (**left**) and asynchronous (**right**) 2D-COS spectra of the polymer–water mixtures after linear offset correction, in the ranges from 0–10% water (*w*/*w*), of PP (**left**) and cellulose (**right**). Note, the intensity scale of the synchronous 2D-COS spectra is the same for all polymers. The remaining 2D-COS spectra are displayed in the Supplementary Materials (Figure S8).

The NIR spectra of polymer–water systems were also analyzed with help of the 2D-COS approach, as it is known to be superior in the deconvolution ability of spectra [41], as well as in elucidating the effects of intermolecular interactions. The exemplary 2D-COS spectra of PP and cellulose are displayed in Figure 7, while the synchronous and asynchronous 2D-COS spectra of all investigated polymers are provided in the Supplementary Materials (Figure S8).

It is immediately noticeable that both systems show a distinctly different correlation pattern. The synchronous 2D-COS plots reveal the presence of only positive cross peaks, which is expected, considering that the investigated sample set features increasing water concentration. In the synchronous 2D-COS of PP in Figure 7 intense diagonal peaks for both water bands are observed, indicating a high magnitude of spectral changes associated with water addition at these wavelengths. Moreover, peak shapes also reflect the broadening of the water bands with increasing water content. The observed cross-peaks on the other hand show the high extent of correlation between both water bands, as a similar increase of intensities of both bands occurs with the addition of water. 

Figure S8 reveals visible interactions of the synthetic polymers with water below 4500 cm^−1^. Furthermore, in the case of the hydrophilic polymers POM and PA, additional interactions of polymer with water are visible in the wavenumber region of 6500–5500 cm^−1^. Interestingly, the biopolymers reveal a completely dissimilar correlation pattern, in contrast to the other investigated polymers. Much less profound correlations are observed for these systems, despite their high hydrophilicity. This might result from a relatively higher content of strongly bound water present in the biopolymer matrix even in nominally similar state of dryness as the other examined polymers. As already mentioned in Section 3.5.2, hydrogen-bonded species lead to lower band intensities in the NIR spectra [26,27]. Therefore, the spectral pattern of the water component changes less radically with increasing water content than it appears for less hydrophilic polymers. In other words, the interaction opportunities that the hydrophilic biopolymer matrix creates for water molecules seemingly shows similarities with the one that molecules of water find in a bulk state. All remaining polymers used in this study, on the other hand, show strong interactions of the polymer vibrations with the water bands. The asynchronous spectra in Figure S8 reveal that the sequence of intensity changes between both water bands is dissimilar for polymers of diverging hydrophilicity. In the case of the non-hydrophilic PP, both water bands show the same behavior. Conversely, for polymers of low hydrophilicity, PS and PVC, the overtone water band reacts more rapidly to the increase in water content. In contrast, for hydrophilic polymers, the overtone band of water reacts less rapidly than the combination water band. The latter effect appears to be less profound for POM and is more decisive for the biopolymers.

### 3.8. Aquagrams

**Figure 8:**
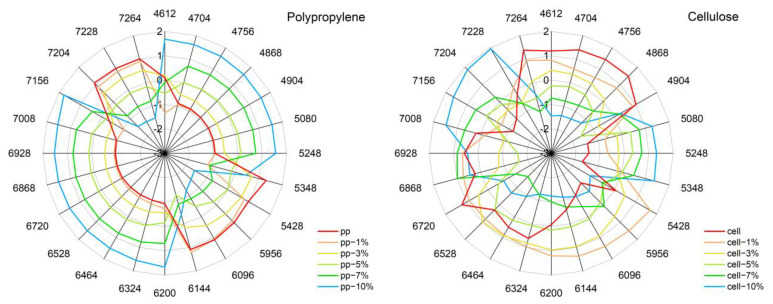
Aquagrams of both water regions for the polymer-water mixtures after SNV and standardization, in the ranges from 0–10% water (*w*/*w*), of PP (**left**) and cellulose (**right**). Aquagrams of all investigated polymers are displayed in the Supplementary Materials (Figure S9).

An aquagram is a unique way for rescaling the spectral intensity at selected key wavenumbers and presenting the data with magnified differences that are less perceptible in absolute scale. For better representation of the polymers, we displayed the normalized spectra of each polymer in both water regions. Wavenumbers of interest were selected by comparison of the transformed polymer spectra; the detailed information about this procedure is given in Methods Section 2.2.6. In Figure 8, the aquagrams obtained for PP and cellulose are displayed as the examples, and the remaining aquagrams of all investigated polymers are provided in the Supplementary Materials (Figure S9). 

While useful for assessing intensity trends of large sets of data at glance, aquagrams are less suited to present an exhaustive cross-section of complex spectral variations. However, when analyzed together with the results provided by the methods discussed in previous sections, a deeper interpretation of the information encoded in aquagrams becomes possible. In general, aquagrams remain in agreement with the information derived from the other methods used in this study, while also revealing unique insights. In the case of hydrophobic to slightly hydrophilic polymers, the water component is highly dominant in the aquagrams, as it can be observed in Figure 8 for PP. At most of the meaningful wavenumbers selected for the aquagrams, a profound increase of the intensity of water bands with rising water content is reflected. Whereas there appear to be spectral regions where the polymer itself has higher contributions to the aquagram than water. Moreover, shifts of the water-dominated areas, i.e., water band shifts, can be easily monitored in the aquagrams. Interestingly, in the case of hydrophilic polymers, the aquagrams become highly complex, reflecting a convoluted spectral pattern associated with the changing water concentration in these systems. The characteristic water bands are not as similar to bulk water in the aquagrams, as they are manifested in the systems constituting more hydrophobic polymers. Hence, aquagrams can immediately identify the systems where a high degree of interaction with water occurs. 

## 4. Discussion

### 4.1. Polymer Hydrophilicity as the Background for the NIR Spectral Trend in Polymer–Water Systems

The concept of hydrophilicity and hydrophobicity is very useful for the comparison of functional groups [42], as well as for capturing the relationship between polymer structure, properties or polymer solubility [9]. It is also frequently used as a physical property for block copolymers [43] and other nanostructures. However, this concept shows its limits when too dissimilar polymers are compared [42]. Despite the concept of hydrophobicity frequently being mentioned in literature, it is still challenging to quantify hydrophobicity in a definitive manner. Polymers are large macromolecules, while the concept of hydrophobicity applies best for single functional groups or small, rigid molecules [9,42].

**Figure 9:**
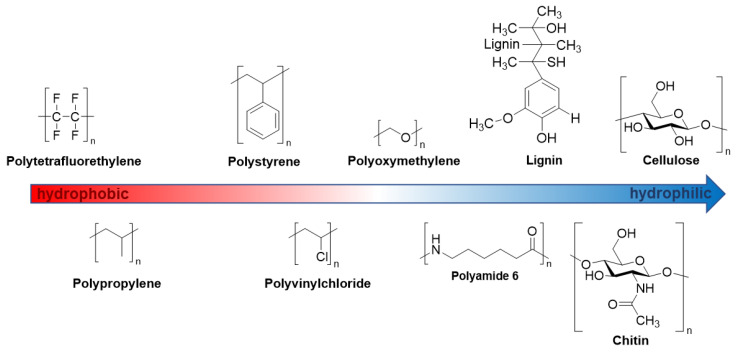
Approximate order of hydrophilicity of the polymers used in this study. From the most hydrophobic (**left**) to the most hydrophilic (**right**) polymers: Polytetrafluoroethylene, polypropylene, polystyrene, polyvinylchloride, polyoxymethylene, polyamide 6, lignin, chitin and cellulose.

The hydrophobicity of a polymer directly influences the interaction with solvents and, thus, the solubility or self-assembly behavior in the solution phase [9,43]. Natural and synthetic polymers feature various hydrophilicity levels and therefore interact differently with water. These interactions distinctly influence the physical properties of water and the polymers [1]. Nonetheless, the polymers used in this study may be approximately ordered with respect to their hydrophilicity as shown in Figure 9 [9,44,45]. This opens the question of whether NIR spectra that are sensitive to intermolecular interactions in a specific way (as discussed in Section 1) can bring new insights into the state of water in a well-defined chemical environment that features a gradually changing hydrophobicity.

### 4.2. General Discussion and Comparison of the Information Derived from Synergistic Methods

Each method used in this study contributes to clarifying the interaction of water with polymers of varying hydrophilicity. The MCR-ALS analysis separates (i.e., deconvolutes) the investigated NIR spectra of polymer–water systems into the spectral components, i.e., water and polymer spectra. The application of this method unveils resolved water curves clearly affected by the interactions of water and polymers. Furthermore, the resolved spectra of water in Figure 6 are surprisingly similar to the regression coefficients in Figure 3. Moreover, distinct band shifts and a dissimilar behavior of both water bands was revealed. The MCR-ALS analysis provides the averaged resolved component spectra of water and polymer from the investigated water–polymer mixtures in the concentration range of 1–10% water in the sample (*w*/*w*). Therefore, this method delivers a centralization of induced changes by the interaction of varying water contents with polymer samples, manifesting in the water bands in the presence of different polymers.

On the other hand, the water difference spectra show more detailed changes correlated to varying water content and dissimilarities among different polymers. Interestingly, this method evidenced the presence of strongly bonded water molecules observed in hydrophobic matrices. Contrarily, the spectra of hydrophilic polymers revealed that rather weakly interacting water species are present in such matrices at low concentrations. Therefore, water difference spectra suggest that the formation of strongly-interacting bulk-like water domains is promoted in hydrophobic matrices. A hydrophilic matrix attracts more water molecules than hydrophobic surfaces. Consequently, hydrophilic polymers create a more competitive environment for binding water molecules, and bulk-like water domains are less easily formed at very low concentration of water in the matrix.

Both methods revealed polymers of low to moderate hydrophilicity, i.e., PP, PS, PVC and POM, to create a special chemical environment for water molecules in NIR. The resolved water spectra reveal trends of spectral changes of the combination and overtone band of water roughly corresponding to the hydrophilicity of the polymer matrix, albeit with specific features associated with the chemical nature of the polymer. Furthermore, a broadened absorption feature towards lower wavenumbers for both water bands appear in hydrophobic polymer matrices. The former effect identifies a weak interaction of water and the polymer, while the latter reveals the presence of strongly interacting water, i.e., self-interacting water. 

Comparing the resolved water difference spectra (Figure S6) with MCR-ALS, deconvolution (Figure S7) reveals that there are three diverse behaviors present among polymers of varying hydrophobicity with water. Firstly, in polymer matrices of very weak hydrophilicity, i.e., PP and PS, water molecules tend to form bulk-like water domains rather than being attracted to the polymer surface. Therefore, in this case, water bands resemble those of pure liquid water. However, for PS, probably additional sterically driven captivation of water molecules is present. The second case is formed for polymers of low or medium hydrophilicity, i.e., PVC and POM, which weakly interact with water. Therefore, also for these samples, additional bulk-like water domains are formed. Thirdly, for hydrophilic polymers which strongly interact with water, i.e., PA, chitin and cellulose, no clear manifestation of bulk water domains can be seen in the spectra. Interestingly, lignin forms an exception in this trend. It should be noted that the actual hydrophilicity of lignin is difficult to estimate owing to its complex structure (Figure 9). Therefore, the molecular environment created for water molecules by lignin may promote a relatively stronger formation of bulk-water domains at low water concentration levels, effectively resembling the features of nominally more hydrophobic polymers. Further investigations are needed to provide insights into this phenomenon; however, these findings reveal a high sensitivity of water towards its chemical environment and attribute it to the interaction of water with the polymer matrices.

Furthermore, the application of the 2D-COS approach revealed that the sequence of intensity changes between both major water bands is dissimilar for polymers of diverging hydrophilicity. In the presence of very weak hydrophilic polymers, i.e., PP, both water bands manifest the same behavior. For polymer matrices of weak hydrophilicity, PS and PVC, the intensity of the overtone water band reacts more rapidly to the increase in water content than it occurs for the combination water band. Contrarily, for hydrophilic polymers, the overtone band of water reacts less rapidly. Moreover, this behavior is less profound for POM and PA, but it is more decisive for highly hydrophilic polymers, i.e., lignin, chitin and cellulose. This effect is also noticeable for biopolymer matrices in water difference spectra. The intensity of the overtone band increases less rapidly with an increasing water concentration in the matrix than is observed for the intensity of the combination band.

By rescaling the intensities of the NIR spectra, aquagrams provide the ability to highlight spectral changes of largely different magnitude, which would be difficult to trace in absolute scale of spectral intensity. Therefore, not only intensity variations of great magnitudes are displayed, but also, small changes in the NIR spectra can be easily monitored, by using aquagrams. While aquagrams are very useful to extract intensity trends occurring in large datasets while displaying those at glance, they are less suited for the comprehensive presentation of spectral variations and their interpretation, e.g., band shifts and changes in band shape cannot be easily followed in this form of presentation. However, when used in combination with other methods of spectral analysis, aquagrams can help in identifying the spectral regions of interest for elucidating the spectral pattern associated with the change in water concentration in the matrix. In this study, the application of aquagrams jointly with the other approaches to analyze the behavior of water in polymer matrices of varying hydrophobicity and chemical nature showed the usefulness of aquagrams for rapid qualitative assessment of the matrix property. Polymer–water systems of weak interaction strength therefore show the profound increase of the intensity of water bands and rather smooth patterns displayed in aquagrams. Contrarily, for hydrophilic polymers, aquagrams become highly convoluted and reflect the complex interaction of the polymer–water systems. Furthermore, water band shifts are immediately noticeable in the aquagrams. Therefore, the aquagrams can, at a glance, reveal the varying complexity of the matrix as it creates different environments for water molecules. This information seems helpful for screening large spectral sets with the purpose of identifying the systems of particular interest for molecular studies of the interactions of water with various chemical environments.

## 5. Conclusions

In this study, we unveiled trends associated with the chemical nature of the polymer and its increasing hydrophilicity, which are specifically manifested in NIR spectra. The results obtained with several independent methods provide confirmatory conclusions, with each method also providing unique findings. The MCR-ALS method and water difference spectroscopy revealed that polymers of varying hydrophilicity manifest three major dissimilar behaviors. Firstly, polymers of very low hydrophilicity feature non-attracting behavior towards water, and therefore, bulk-like water domains are formed more easily in the sample. Secondly, the polymers of low or medium hydrophilicity weakly interact with water, and additionally, bulk-like water domains are formed. Thirdly, hydrophilic polymers strongly interact with water; therefore, no clear evidence of bulk water domains is present in the NIR spectra of polymer–water systems. Of particular interest is the dissimilar spectral manifestation of both major water bands, located at ca. 6900 cm^−1^ and 5200 cm^−1^ (*ν*_s_ + *ν*_as_ and *ν*_as_ + *δ*) in the presence of diverse polymers. Some polymers show simultaneous blue- and redshifts for both major water bands. Furthermore, wavenumber shifts are much more pronounced for the overtone water band (6900 cm^−1^) than they are for the combination band (5200 cm^−1^).

The 2D-COS analysis revealed that the sequence of intensity changes of the water bands is dissimilar for polymers of varying hydrophilicity. While for polymers of weak hydrophilicity, the overtone water band reacts more rapidly to the increase in water content than the combination band; this trend is opposite for hydrophilic polymers. The experimental findings by difference spectroscopy proved that even highly hydrophobic polymers (e.g., PTFE) interact with water, and these interactions manifest themselves in the water component of the NIR spectra. Hydrophilicity, therefore, is not exhaustive enough to describe the interaction of a polymer with water. Taking into account the chemical specificity of the matrix in describing spectral effects of the water–substance interactions is necessary for successful removing of the water contributions in NIR spectra. The analysis of the polymer–water mixtures also confirmed that the sensitivity of water towards its chemical environment is a major factor clearly manifested in NIR spectra. Moreover, with increasing hydrophilicity of the matrix, in NIR spectra the amplitude and complexity of spectral variations resulting from water–matrix interactions are enhanced. The 2D-COS investigations confirmed that strong hydrogen-bonding leads to a diminished band intensity of the interacting species in NIR spectra [26,27].

Finally, aquagrams are a unique way for rescaling the data and showing wavelength-specific phenomena. Water band shifts are immediately noticeable in the aquagrams. When compared with the other methods used in this study, the usefulness of aquagrams for rapid assessment of the interaction strength of water with the sample matrix was shown. Furthermore, when compared with the outcomes of the MCR-ALS procedure, aquagrams seem capable of highlighting effects, which could not be easily derived in difference spectra.

## Figures and Tables

**Table 1:** Wavenumber assignments of relevant polymer and water groups [39].

Wavenumber/cm^−1^	Assignment	Polymer/Water
10,000–9000	3 ν (OH); hydrogen-bonded	
8600–8200 [21,39] 8250 [21]	3 ν (CH_3_ [21,39], CH_2_ [39]) 2 ν + 2 δ (CH_3_, CH_2_)	all
7200–7000	2 ν (free OH) 2 ν + δ (CH_3_, CH_2_)	Lig, Chi, Cell all
7200–6800 [25]	ν_s_ + ν_as_ (OH)	water
7000–6200	2 ν (OH); hydrogen-bonded ν (OH) + ν (CH)	all
6900 [40]	2 ν CH + δ CH	all
6700–6500	2 ν (NH); free	PA, Chi
6600–6300	2 ν (NH); hydrogen-bonded	
6500	2 ν (OH); carbohydrates, polyphenols, …; hydrogen-bonded	Lig, Chi, Cell
6200	ν (CH_3_, CH_2_)	all
6000–5600	2 ν (CH_3_, CH_2_) ν_s_ + 2 δ (CH_3_, CH_2_)	
5300–5000 [25] 5200 [11,39,40]	ν_as_ + δ (OH)	water
5280 [11]	Hydrogen-bonded water	water
5190 [11,39]	ν_as_ + δ (OH) water molecule trapped in Polymer	Cell + water
5150 [28]	ν_as_ + δ (OH); water molecule trapped in Polymer	PA + water
4900–4600	ν + δ (NH)	PA, Chi
4500	ν (CH_3_, CH_2_)	all
4400–4200	ν + δ (CH_3_, CH_2_)	all

ν—stretching; δ—bending vibration; ν_s/as_—symmetric/asymmetric; 2—first overtone; 3 - second overtone.

**Table 2:** By difference spectroscopy, we revealed wavenumber shifts of the overtone and combination water band in the concentration range of 1–10% of water (*w*/*w*) for the investigated polymers. Note, wavenumber shifts for lignin are given in brackets, because the experimental data may be considered less reliable.

Polymer	Shifts for Overtone Water Band/cm^−1^	Shifts for Combination Water Band/cm^−1^
Polypropylene	6796–6836	5180/not shifted
Polystyrene	6812–6852	5248–5176
Polyvinylchloride	6808–6842	5256–5180
Polyoxymethylene	6822–6826	5228–5204
Polyamide 6	6828–6840	5140–5164
(Lignin)	(7084–7064)	(5224–5208)
Chitin	7048–7024	5176–5164
Cellulose	7100–7120	5180–5172

## Data Availability

Not applicable.

## Supplementary Materials

The following supporting information can be downloaded at: https://www.mdpi.com/article/10.3390/molecules27185882/s1. Figure S1: PCA Scores; Figure S2: MLC Dendrograms; Figure S3: PLSR Scores; Figure S4: PLSR Regression Coefficients; Figure S5: PLSR Predicted vs. Reference; Figure S6: Water Difference Spectra; Figure S7: MCR-ALS; Figure S8: 2D-COS; Figure S9: Aquagrams.

## Sample Availability

Samples are not available.
