# Supplementary material for: Investigation of Water Interaction with Polymer Matrices by Near-Infrared (NIR) Spectroscopy
Source: Molecules. 2022 Sep 10;27(18):5882. doi: 10.3390/molecules27185882 (PMC9504856; doi:10.3390/molecules27185882)
Supplement: Supplementary file 1 [file molecules-27-05882-s001.zip › molecules-1887176-supplementary.pdf]

PCA Scores

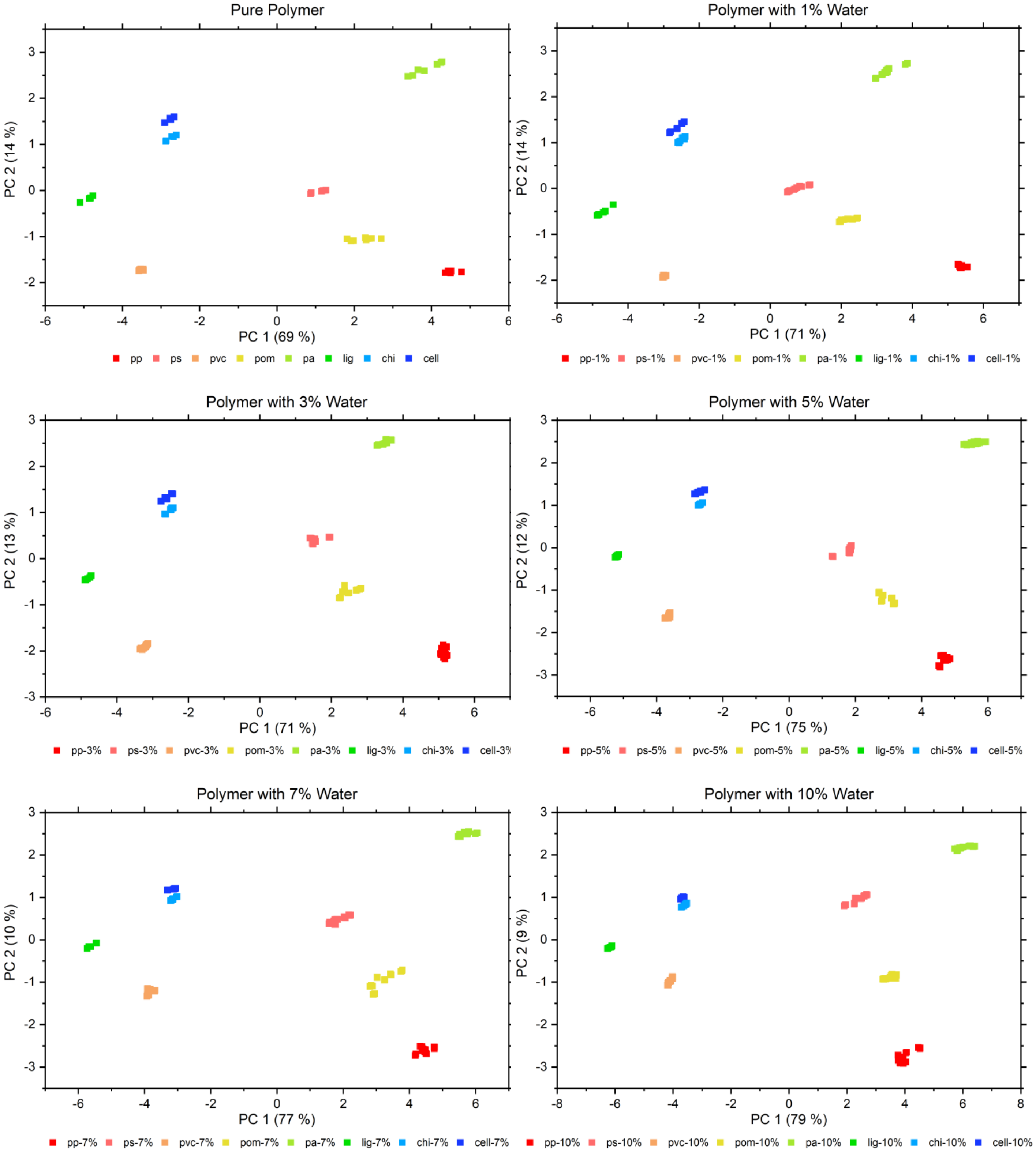

Figure S1. PCA scores for the pure polymers and the polymer-water mixtures in the range of 1-10 % water added to the polymer (w/w), of all investigated polymer-water mixtures after linear offset correction. The scores plots are ordered according to increasing water content, with the pure polymer (upper left corner), to the highest water content added to the polymer, 10% of water (lower right corner).

MLC Dendrograms

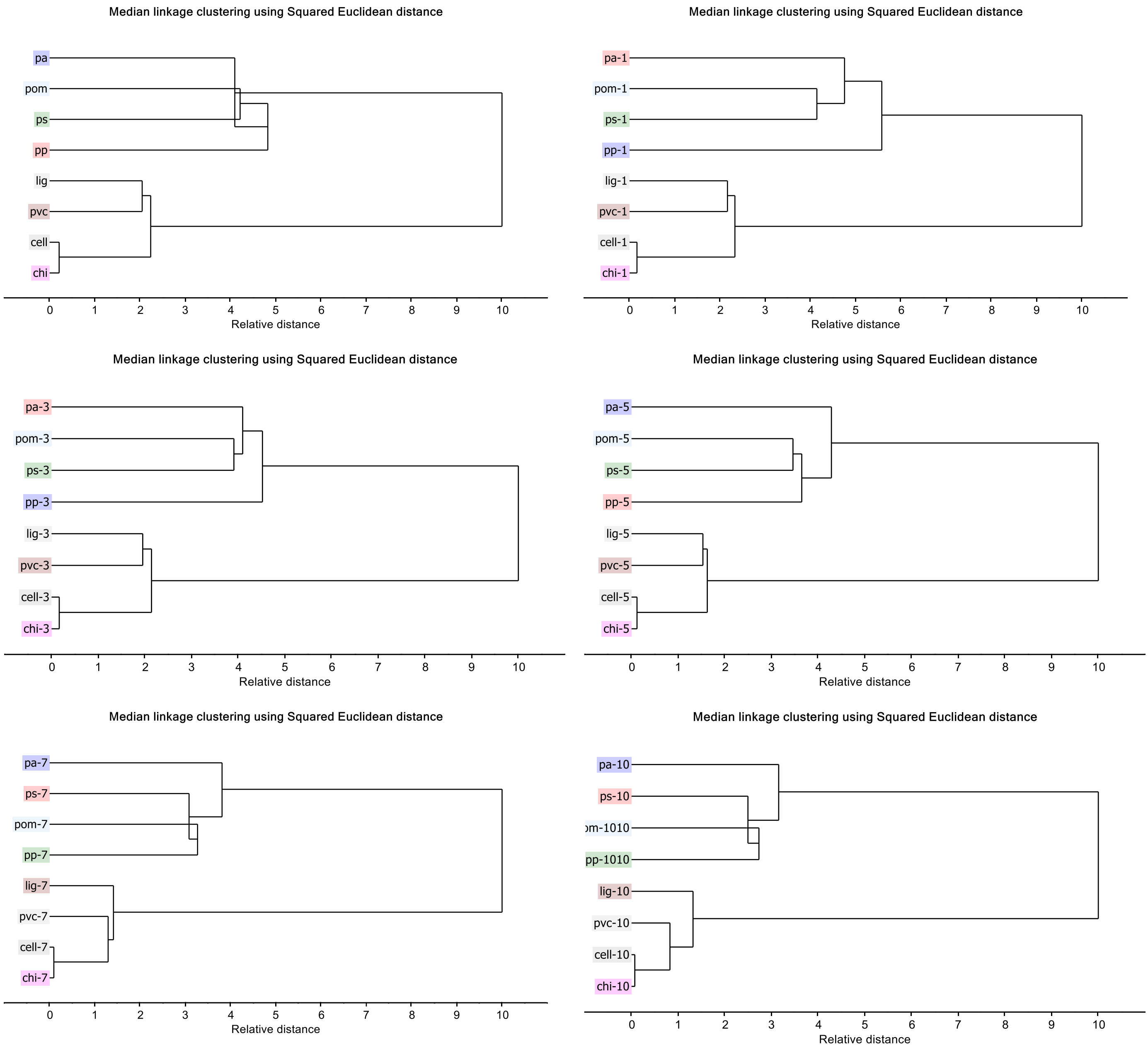

Figure S2. MLC dendrograms for the pure polymers and the polymer-water mixtures in the range of 1-10 % water added to the polymer (*w*/*w*), of all investigated polymer-water mixtures after linear offset correction**.** The dendrograms are ordered according to increasing water content, with the pure polymer (upper left corner), to the highest water content added to the polymer, 10% of water (lower right corner).

PLSR Scores

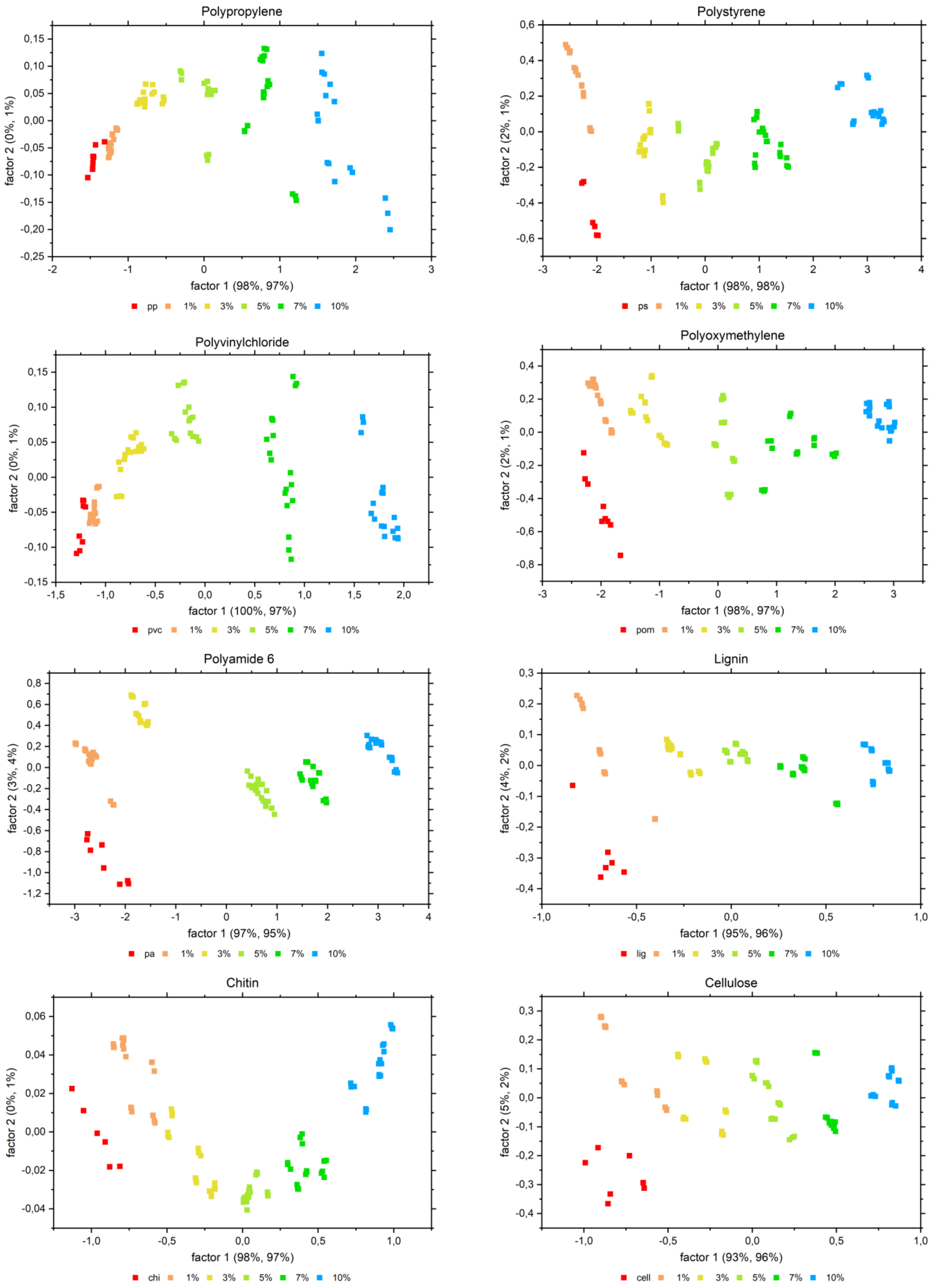

Figure S3. PLSR scores of all investigated polymer-water mixtures after linear offset correction, in the range of 0-10 % water (*w*/*w*). The polymers are ordered according to increasing hydrophilicity, with the least hydrophilic polymer, polypropylene (upper left corner), to the most hydrophilic polymer, cellulose (lower right corner).

PLSR Regression Coefficients

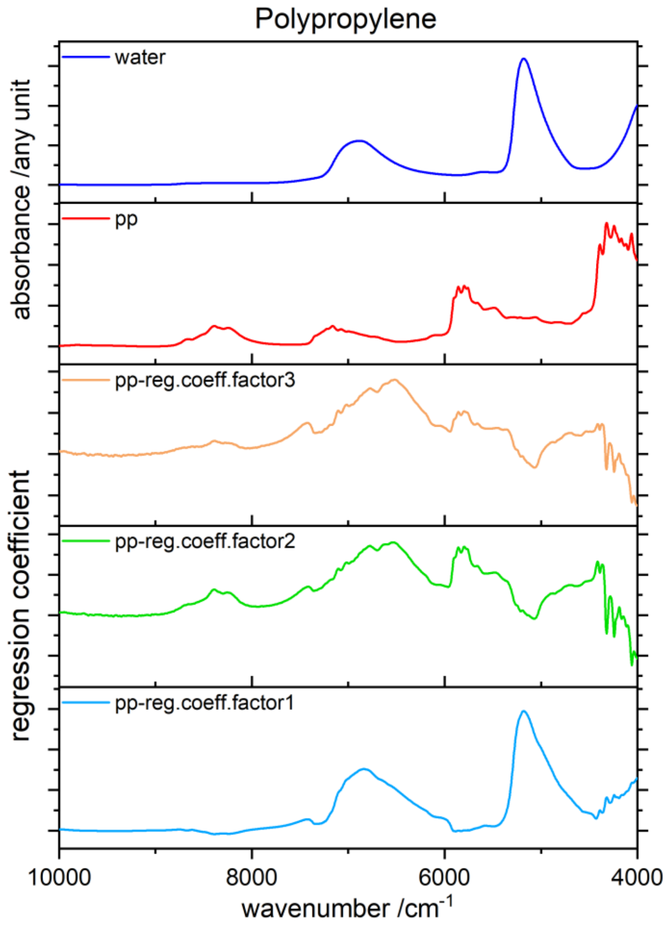

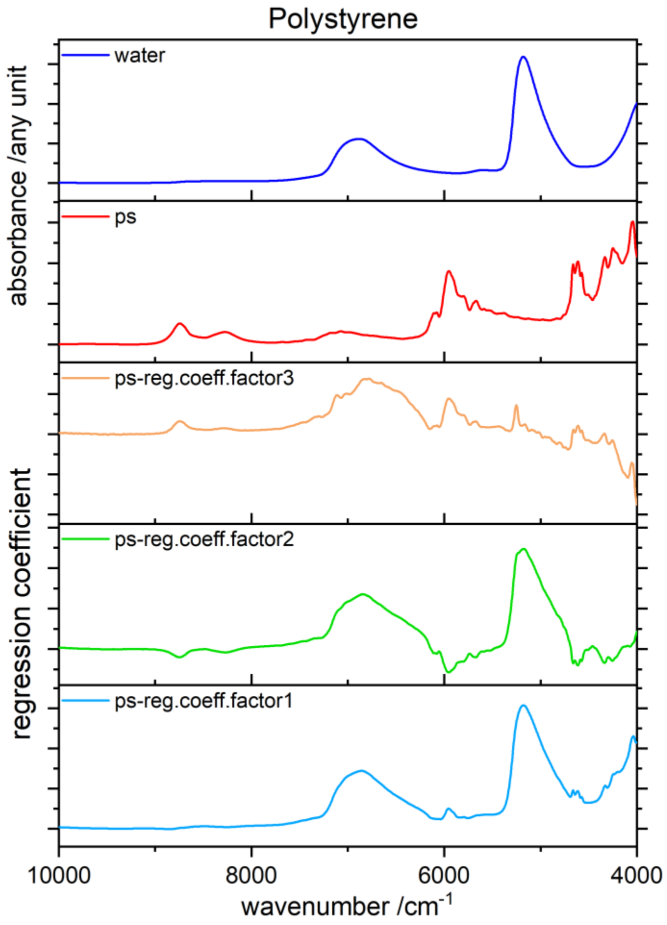

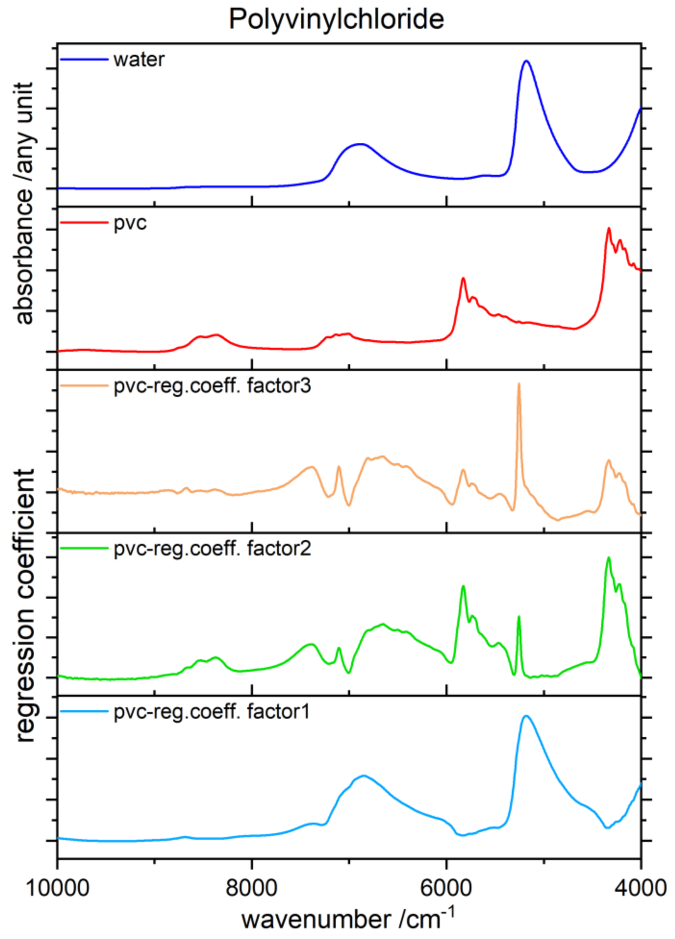

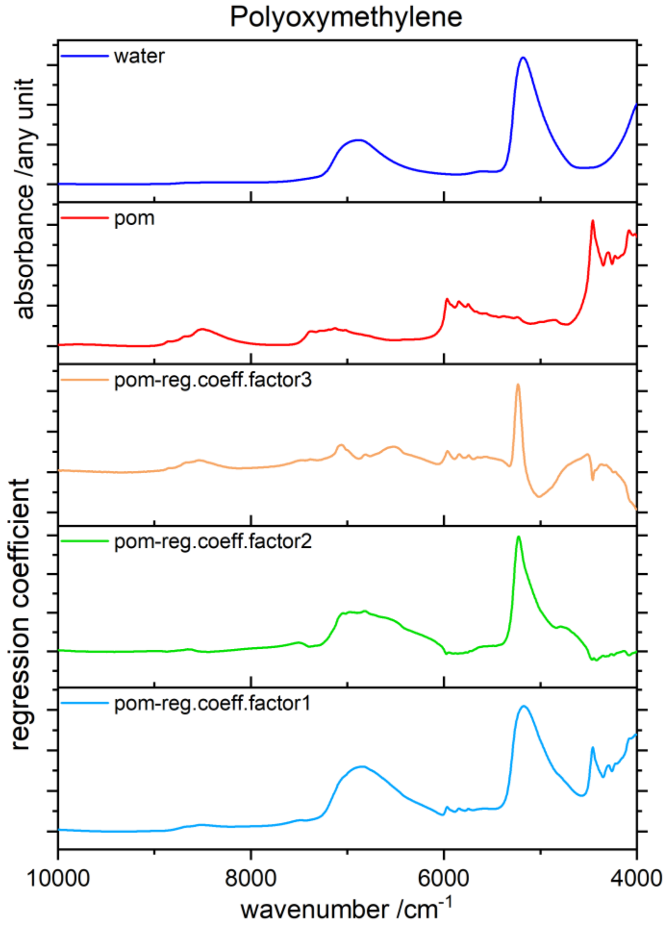

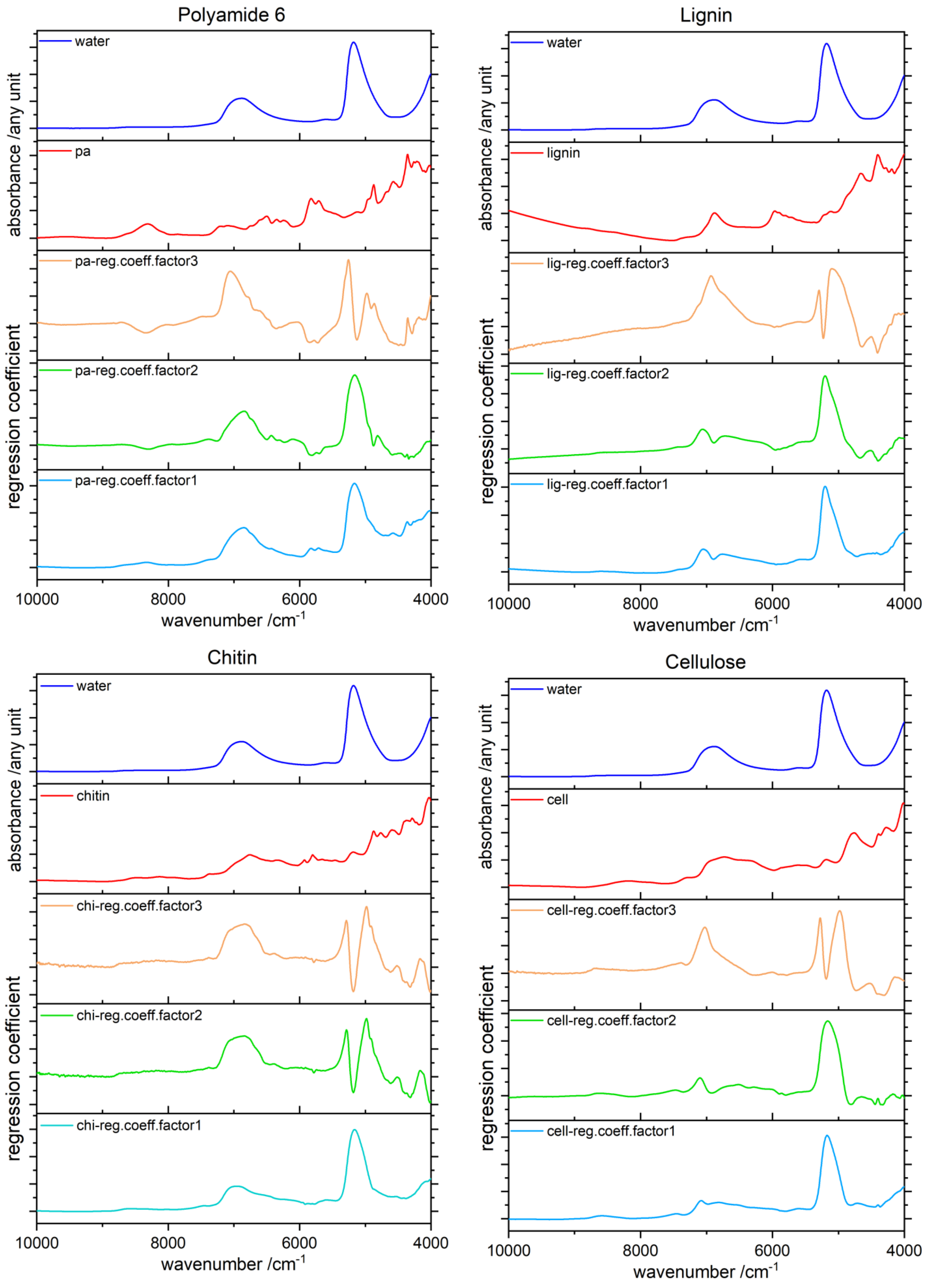

Figure S4. PLSR regression coefficients for the factors 1-3 of all investigated polymer-water mixtures after linear offset correction, in the range of 0-10 % water (*w*/*w*). The polymers are ordered according to increasing hydrophilicity, with the least hydrophilic polymer, polypropylene (upper left corner), to the most hydrophilic polymer, cellulose (lower right corner).

PLSR Predicted vs. Reference

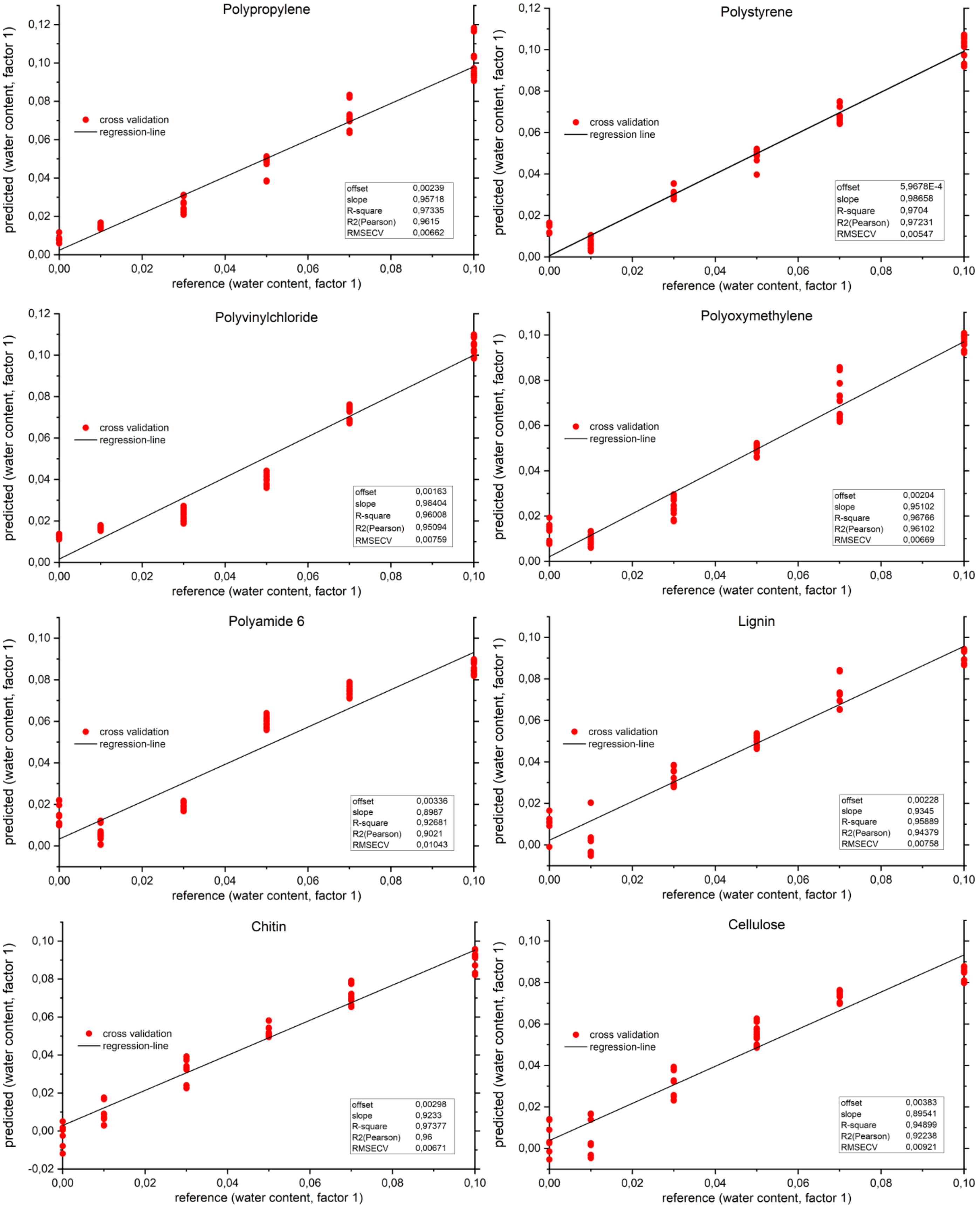

Figure S5. PLSR predicted vs. reference of all investigated polymer-water mixtures after linear offset correction, in the range of 0-10% water (*w/w*). The polymers are ordered according to increasing hydrophilicity, with the least hydrophilic polymer, polypropylene (upper left corner), to the most hydrophilic polymer, cellulose (lower right corner).

Water Difference Spectra

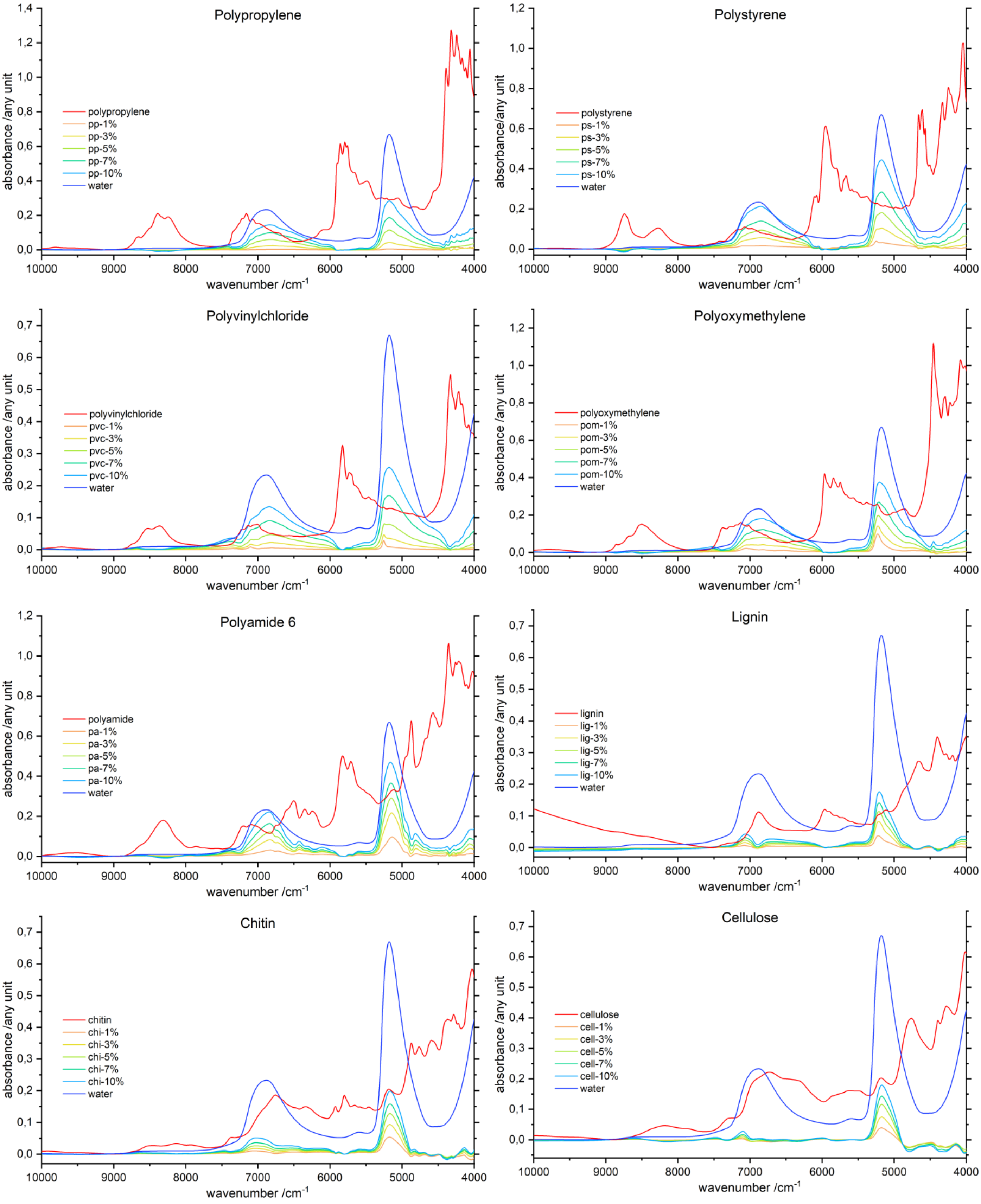

Figure S6. Water difference spectra of all investigated polymer-water mixtures after linear offset correction and subtraction of the polymer spectra, in the range of 1-10 % (*w*/*w*), with respectively the pure water (dark blue) and polymer (red) spectra for comparison. The polymers are ordered according to increasing hydrophilicity, with the least hydrophilic polymer, polypropylene (upper left corner), to the most hydrophilic polymer, cellulose (lower right corner).

MCR-ALS

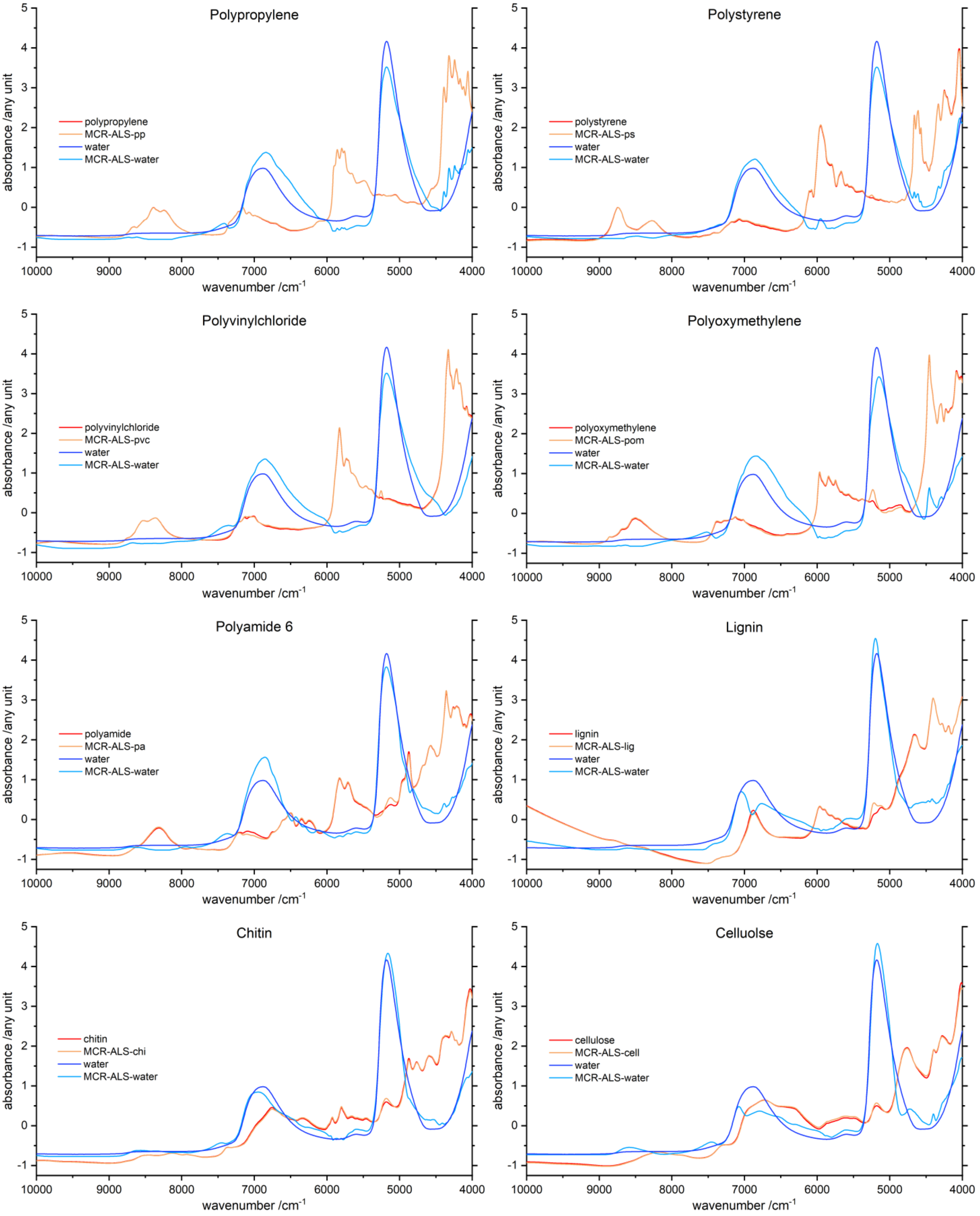

Figure S7. MCR-ALS polymer (orange) and water spectra (light blue) of all investigated polymers, additionally the NIR absorbance spectra of the pure polymer (red) and pure water (dark blue). The reference spectra as well as the resolved curves were normalized using SNV transformation. The polymers are ordered according to increasing hydrophilicity, with the least hydrophilic polymer, polypropylene (upper left corner), to the most hydrophilic polymer, cellulose (lower right corner).

2D-COS

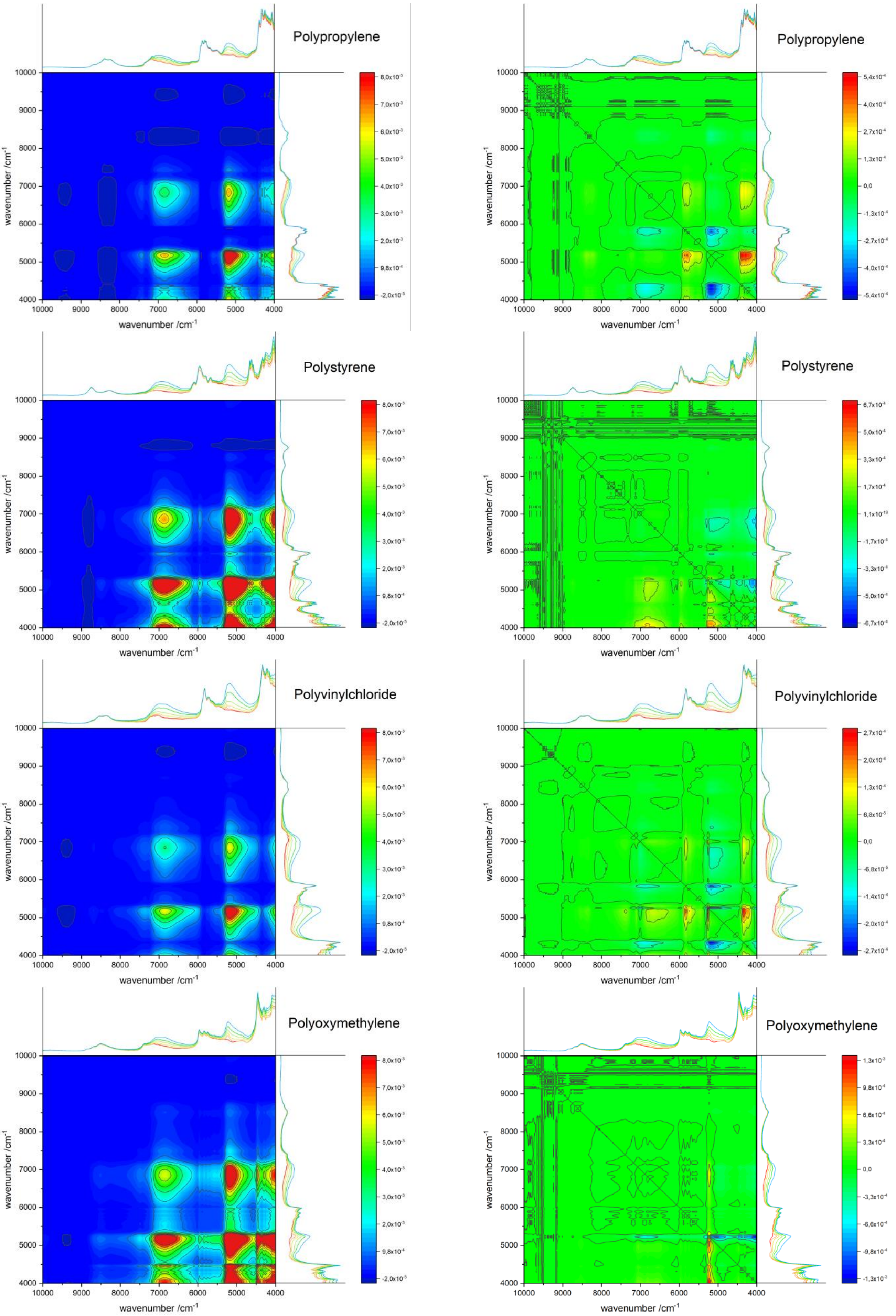

Polypropylene
Polypropylene
Polystyrene
Polystyrene
Polyvinylchloride
Polyvinylchloride
Polyoxymethylene
Polyoxymethylene
wavenumber /cm-1
wavenumber /cm-1

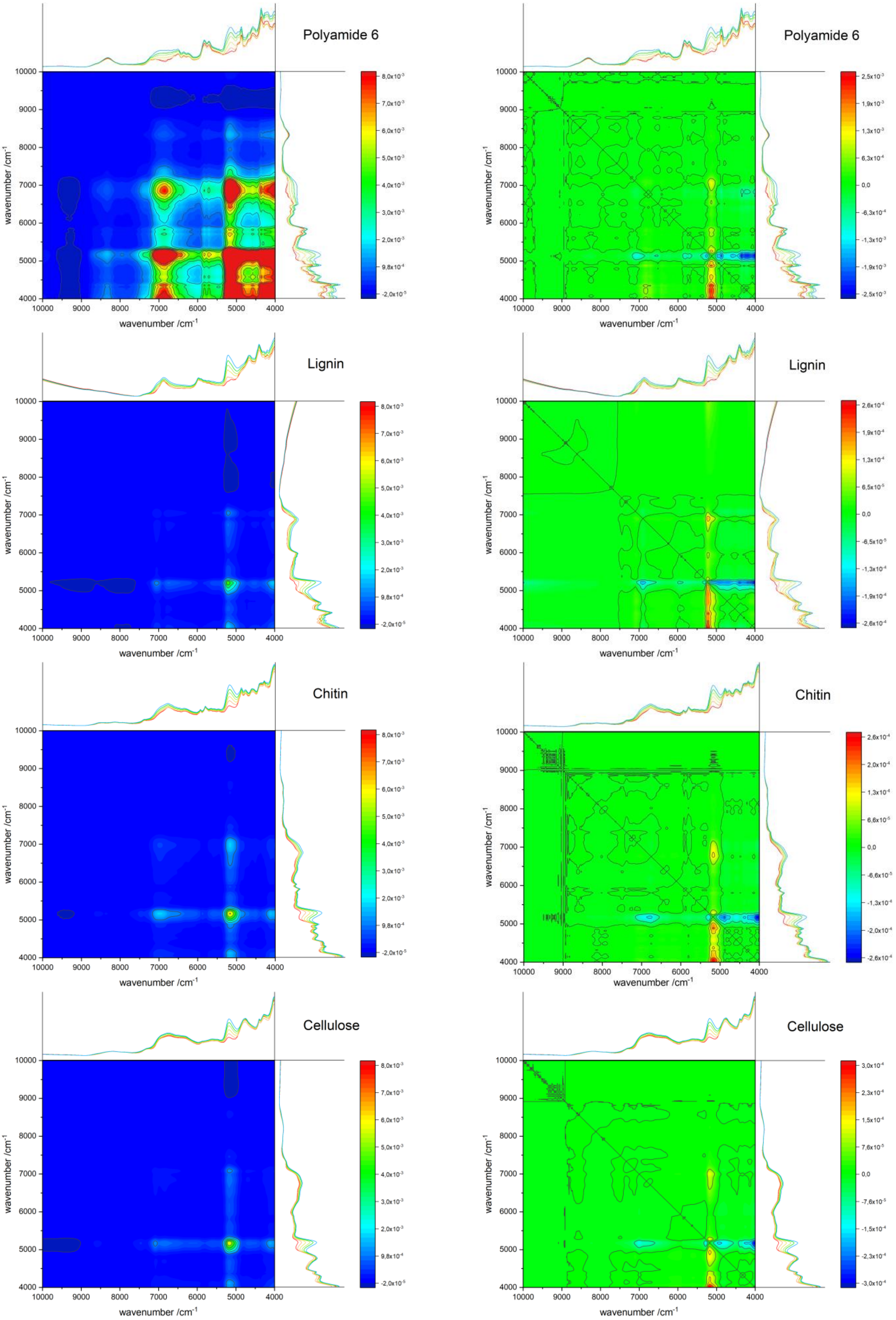

Figure S8. Synchronous (left) and asynchronous (right) 2D-COS spectra of the polymer-water mixtures after linear offset correction, in the ranges from 1-10 % water (*w*/*w*), of all investigated polymers. Note, the intensity scale of the synchronous 2D-COS spectra is the same for all polymers. The polymers are ordered according to increasing hydrophilicity, with the least hydrophilic polymer, polypropylene (top), to the most hydrophilic polymer, cellulose (bottom).

Aquagrams

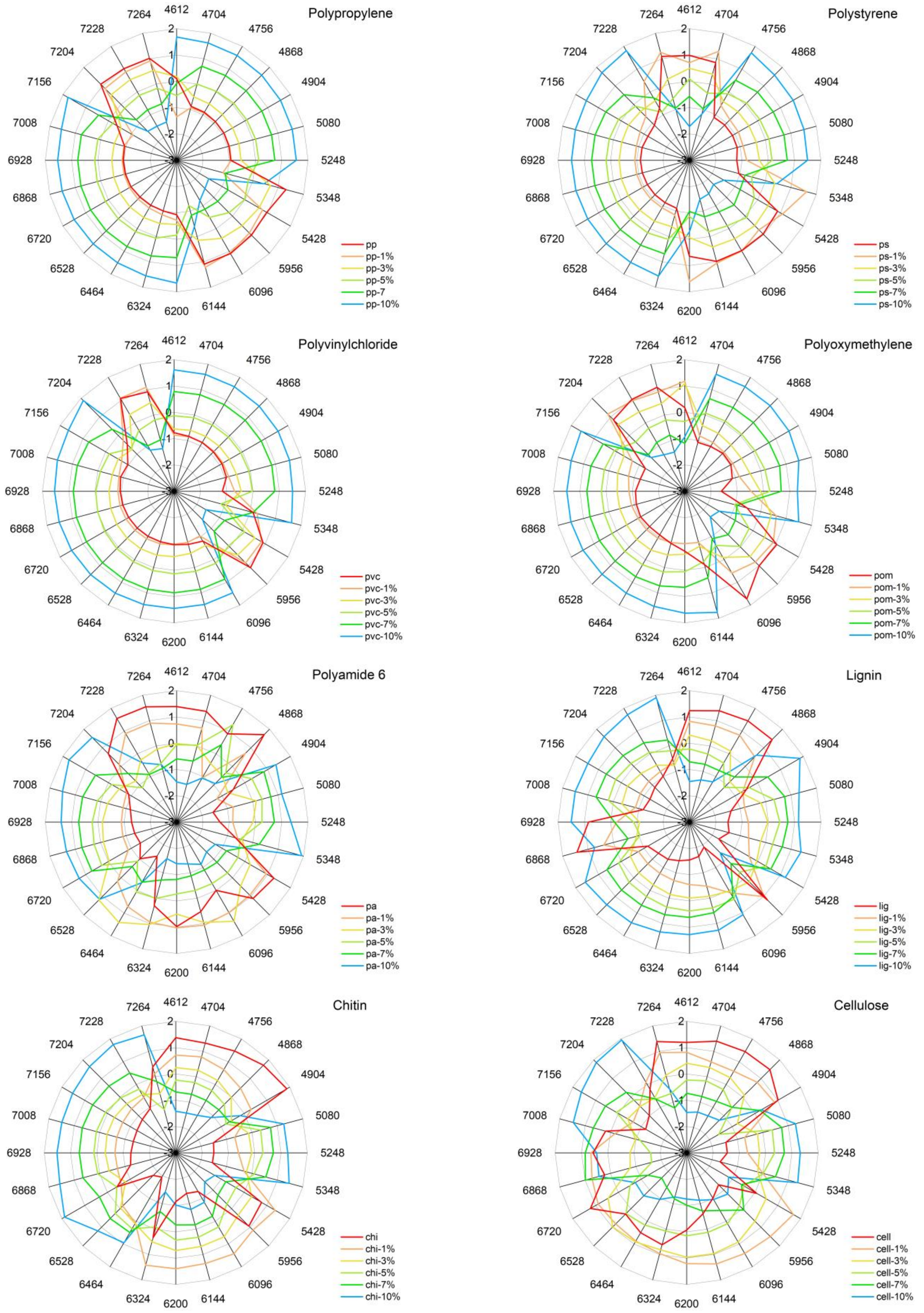

Figure S9. Aquagrams of the both water regions for the polymer-water mixtures after SNV and standardization, in the ranges from 0-10 % water (*w*/*w*). The polymers are ordered according to increasing hydrophilicity, with the least hydrophilic polymer, polypropylene (upper left corner), to the most hydrophilic polymer, cellulose (lower right corner).
